# Implementation of the Crisis Resolution Team model in adult mental health settings: a systematic review

**DOI:** 10.1186/s12888-015-0441-x

**Published:** 2015-04-08

**Authors:** Claire Wheeler, Brynmor Lloyd-Evans, Alasdair Churchard, Caroline Fitzgerald, Kate Fullarton, Liberty Mosse, Bethan Paterson, Clementina Galli Zugaro, Sonia Johnson

**Affiliations:** 1 Division of Psychiatry, UCL, London, Charles Bell House, 67-73 Riding House Street, London, W1W 7EJ UK; 2 Department of Psychology, University of Bath, Claverton Down, Bath, North East Somerset BA2 7AY UK

**Keywords:** Crisis resolution team, Home treatment team, Crisis assessment and treatment team, Mental health, Service implementation, Good practice, Systematic review

## Abstract

**Background:**

Crisis Resolution Teams (CRTs) aim to offer an alternative to hospital admission during mental health crises, providing rapid assessment, home treatment, and facilitation of early discharge from hospital. CRTs were implemented nationally in England following the NHS Plan of 2000. Single centre studies suggest CRTs can reduce hospital admissions and increase service users’ satisfaction: however, there is also evidence that model implementation and outcomes vary considerably. Evidence on crucial characteristics of effective CRTs is needed to allow team functioning to be optimised. This review aims to establish what evidence, if any, is available regarding the characteristics of effective and acceptable CRTs.

**Methods:**

A systematic review was conducted. MEDLINE, Embase, PsycINFO, CINAHL and Web of Science were searched to November 2013. A further web-based search was conducted for government and expert guidelines on CRTs. We analysed studies separately as: comparing CRTs to Treatment as Usual; comparing two or more CRT models; national or regional surveys of CRT services; qualitative studies of stakeholders’ views regarding best practice in CRTs; and guidelines from government and expert organisations regarding CRT service delivery. Quality assessment and narrative synthesis were conducted. Statistical meta-analysis was not feasible due to the variety of design of retrieved studies.

**Results:**

Sixty-nine studies were included. Studies varied in quality and in the composition and activities of the clinical services studied. Quantitative studies suggested that longer opening hours and the presence of a psychiatrist in the team may increase CRTs’ ability to prevent hospital admissions. Stakeholders emphasised communication and integration with other local mental health services; provision of treatment at home; and limiting the number of different staff members visiting a service user. Existing guidelines prioritised 24-hour, seven-day-a-week CRT service provision (including psychiatrist and medical prescriber); and high quality of staff training.

**Conclusions:**

We cannot draw confident conclusions about the critical components of CRTs from available quantitative evidence. Clearer definition of the CRT model is required, informed by stakeholders’ views and guidelines. Future studies examining the relationship of overall CRT model fidelity to outcomes, or evaluating the impact of key aspects of the CRT model, are desirable.

**Trial registration:**

Prospero CRD42013006415.

**Electronic supplementary material:**

The online version of this article (doi:10.1186/s12888-015-0441-x) contains supplementary material, which is available to authorized users.

## Background

Crisis Resolution and Home Treatment Teams (CRTs) serve adults experiencing an acute mental health crisis who are otherwise likely to require hospital admission. CRTs aim to provide rapid assessment, to treat service users at home where possible, and to facilitate early discharge from hospital [1]. They offer an alternative to hospital care with the aim of treating people ‘in the least restrictive environment with the minimum disruption to their lives’ ([2] p.11). CRTs typically aim to offer 24-hour access, intensive support and a “gatekeeping” function (controlling access to inpatient beds and assessing suitability for home treatment before admission) [1].

### CRTs in England

Provision of CRTs in all catchment areas became mandatory in England in 2000 under the National Health Service (NHS) Plan [3]. Nationwide introduction of this model was achieved over the next few years, but with variable adherence to the Department of Health’s original guidance [4]. A national survey of CRTs in 2005/6 found that only 40% of teams described themselves as fully established according to the Department of Health’s [3] guidance, with a third of teams not involved in gatekeeping, and just over a half of teams offering a 24-hour, seven-day-a-week home visiting service [4]. CRT availability is no longer mandatory in England, but the model continues to be prominent: national guidance on service delivery strongly recommends CRTs as a central part of acute service pathways [5,6].

### Impact of CRT implementation

Some single centre studies [7,8], including a randomised trial of CRTs [9], provide evidence that CRTs can reduce the number of hospital admissions, and thus also cut the cost of services [10]. Some naturalistic studies have suggested that CRTs can increase service users’ satisfaction with acute care [8,9,11,12]. However, overall reductions in admissions have not been reported everywhere where CRTs have been introduced (for example such reductions were not found in a team in Wales, [13], and national data do not indicate a clear overall effect in reducing admissions [14,15]. Some service users and carers report unsatisfactory experiences of CRT care [16,17]. A higher rate of suicide on CRT caseloads than in acute inpatients has also recently been reported, with concerns raised that risk management may be less than optimal in some teams [18]. Thus evidence suggests that the implementation of the CRT model in England currently does not consistently achieve the intended aims, while implementation of the model also appears to vary greatly in Norway, the other country where CRT introduction has been national policy [19]. There is a need for evidence on how best to implement the CRT model. This should include specification of the organisational structures, specific interventions and ways of working that are likely to optimise outcomes, and the development of methods for assessing service quality and for improving implementation [1].

Previous systematic reviews of CRTs have focussed on whether CRTs are effective, rather than exploring the characteristics that influence their effectiveness. Findings were of increased service user and/or carer satisfaction rates for CRTs versus standard care [20-22]; reduced hospital bed use following introduction of CRT care [23]; and reduced inpatient admissions but inconclusive effect on compulsory admissions [21 in Germany] [20,24]. The specific effectiveness of CRTs for people with borderline personality disorders [25] or for older people [24] is unclear from current evidence.

### Aims and scope of study

Although previous papers have reviewed the effectiveness of CRTs, no review to our knowledge has systematically collected qualitative and quantitative evidence and views regarding key organisational principles and critical components of CRT services. Therefore, this study aims to systematically review randomised and non-randomised comparison studies and national surveys of CRT services, qualitative studies of CRT stakeholders’ views, and national and expert guidelines relating to the implementation of CRTs. We aim to investigate:

i. What characteristics of CRTs are associated with positive outcomes in empirical evaluations of CRT services?
ii. What do service users, carers and staff identify in qualitative studies and surveys and quantitative questionnaires as important elements influencing CRT service quality?
iii. What recommendations do government agencies and non-statutory organisations and experts make regarding CRT service delivery and organisation?

The review follows the Preferred Reporting Items for Systematic Reviews and Meta-analyses (PRISMA statement) [26] and follows guidance from the Centre for Reviews and Dissemination [27] on conducting narrative synthesis. A PRISMA checklist for this review is provided in Additional file 1.

## Methods

### Protocol and registration

The study is registered with PROSPERO international prospective register of systematic reviews at the Centre for Reviews and Dissemination, University of York; registration number CRD42013006415. The protocol can be found online [28].

### Inclusion criteria

#### Services

We included studies of CRTs that offer intensive home treatment for a brief period (typically a month or less on average) to adults with acute mental health problems who would otherwise be admitted to hospital. We included specialist services established for crisis care and integrated services with a clear crisis function. For quantitative studies, comparison treatment as usual (TAU) groups were specialist mental health services that provide multi-disciplinary community-based care (such as UK Community Mental Health Teams).

We excluded studies of intensive home treatment services which offered on-going rather than brief care (such as Assertive Community Treatment teams). In order to assess the impact of CRTs in a contemporary mental health system involving secondary care community mental health teams, we also excluded studies comparing CRT services to treatment as usual where the latter involved only inpatient care or outpatient appointments with a psychiatrist.

#### Participants

At the participant level, the inclusion criterion was that CRTs serve adults with acute mental health problems who would otherwise be admitted to hospital. Studies including older age adults were included if the participants had a functional mental illness rather than an organic mental disorder.

Studies primarily including participants under the age of 16 were excluded.

#### Types of study

The following types of study were included:

1. Quantitative studies of any type comparing outcomes between two or more CRTs with different characteristics or service content.
2. Quantitative studies of any type comparing a CRT service with another type of service or treatment as usual (in order to explore differences in CRT characteristics between studies where the CRT is found to have an association with improved outcomes and studies where there was no effect).
3. National or regional level surveys of CRTs which report associations between service characteristics and outcomes.
4. Qualitative interviews, focus groups or surveys (some also including quantitative questionnaires) of stakeholders’ views (service users, carers and staff) regarding elements of good CRT services.
5. Published guidelines from statutory agencies or non-statutory organisations with responsibility for policy and health services in England, which provided recommendations regarding CRT service delivery and organisation, often based on the views of an expert panel or a panel containing experts and stakeholder group representatives.

In anticipation of few randomised trials being found, studies in categories 1) and 2) were not restricted by methodology: randomised controlled trials and also natural experiments with pre- and post- comparisons and natural experiments with parallel groups were eligible for inclusion.

Studies written in languages other than English were not excluded. Studies conducted up to the time of the last search were included, and there was no time limit specified.

### Search strategy

An electronic database search using MEDLINE, Embase, PsycINFO, CINAHL and Web of Science was conducted using the search terms in Additional file 2: Table DS1 (last search conducted in November 2013). Key words related to concepts of “crisis resolution” and “home treatment” were combined with MeSH terms from the PubMed database and Subject Headings from the PsycINFO database. We did not make restrictions using limit functions.

A web-based search of government and expert organisation guidelines for England regarding CRTs was conducted using the search terms and web resources reported in Additional file 2: Table DS2. Google was also searched.

The title and abstract of all retrieved studies were scanned independently by two reviewers (AC, BLE, CF, BP or CW). The full text of potentially eligible papers was retrieved and decisions about inclusion made by two reviewers (AC, BLE, CF, BP or CW). We screened the reference lists of key papers. Any disagreement regarding inclusion was resolved through discussion or, where necessary, with reference to another reviewer (SJ).

### Data extraction

A data extraction form was used to code and record relevant data from each included study. Data extraction was carried out by a member of the review team (BLE, CF, LM, BP, CW or CGZ) and checked by another member of the team; with discrepancies resolved in consultation with another reviewer (SJ). Information was extracted from included studies on:

1. Study characteristics: type of study; setting; participant numbers and characteristics (for quantitative studies); duration of study and outcomes assessed
2. Results: outcomes and significant findings from quantitative studies; themes and recommendations from stakeholder interviews and guidelines
3. CRT service characteristics: for quantitative studies comparing two CRT service models, we reported the differences between services being studied; for studies of CRTs versus standard care, we reported characteristics of CRTs identified in statutory guidance for England [2] including 24 hour service, gatekeeping function staffing levels, multi-disciplinary team, (defined here as including at least one other professional group in addition to nurses and psychiatrists), medical staffing in team, duration of care and early discharge function to support prompt discharge from hospital.

We contacted authors to ask for any of this information not available from published papers.

### Quality of individual studies

Quality was assessed using the Mixed Methods Appraisal Tool (MMAT) [29]. The tool is applicable to quantitative, qualitative and mixed methods primary studies. We did not exclude papers from the review on account of low quality scores, but quality scores were reported and considered in the narrative synthesis of the evidence. The MMAT quality scoring scale ranges from 0 (low quality) to 4 (high quality). The MMAT has been pilot tested for reliability in systematic reviews [30]. Ratings are specific to particular methodologies, and are based on control of confounding factors; completeness of outcome data; minimisation of selection bias; representativeness of sample; appropriateness of measures; response and withdrawal rates; appropriateness of study design to answer the research questions; and consideration of limitations.

### Synthesis of results

We conducted a narrative synthesis to integrate findings from studies of all methodologies (quantitative, qualitative and mixed methods). The synthesis was structured around the characteristics of CRTs, important elements influencing CRT service quality, and recommendations for CRT service delivery and organisation. Quantitative synthesis of results from quantitative studies was not considered appropriate because of the heterogeneity of types of study, outcomes measured, service settings and characteristics.

## Results

### Study selection

**Figure 1:**
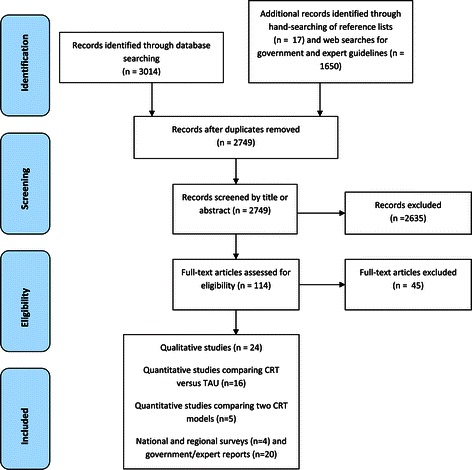
**Study selection flow diagram.**

The Study Selection flow Diagram - Figure 1 - shows the selection and screening of papers to include in the review. After removing duplicates, the database search yielded 2749 studies. The web-based search for expert and government guidelines yielded 1650 papers/reports. After screening, 69 studies and documents were identified for inclusion in the review.

### Study characteristics

The 69 papers included in the review comprised:

1. Comparisons of two CRT models (Table 1) (n = 5) [31-35]: Natural experiments, three with pre-post comparisons. Studies were published between 1994 and 2011; three were set in the UK, one in USA and one in Australia. Outcomes assessed were admission rates, health status at discharge, and service user and carer satisfaction.
2. CRTs versus standard care (Additional file 3: Table DS3) (n = 16) [8,9,12,13,36-47]: Two studies were randomised controlled trials, three were non-randomised (naturalistic) two-group comparison studies; and 11 were naturalistic pre-post comparison studies. Two studies were Australian, one German, one American, and 12 British; studies were published between 1993 and 2011. Primary outcomes in the included studies were hospital admission rates and service user satisfaction ratings. Conclusions were drawn regarding the characteristics of CRTs in these studies in relation to their outcomes.
3. CRT national surveys (Additional file 3: Table DS4) (n = 4) [7,14,19,48]: Two papers reported one UK national CRT survey; two papers reported one Norwegian national survey. The UK survey was first published in 2006; the Norwegian survey in 2011.
4. CRT stakeholder qualitative interviews and quantitative surveys (Additional file 3: Table DS5) (n = 24) [4,16,17,49-69]: Twelve studies included service users as participants, five included carers, and twelve included CRT staff. In 15 studies, individual interviews were conducted in person (seven semi-structured, two structured, six not reported); one involved focus groups and eight involved data collection via online surveys, postal questionnaire or phone interview. The studies included between 1 and 177 CRTs, and between 7 and 471 participants. 17 studies were set in the UK; two each in Australia and Norway; and one each in France, Canada and The Republic of Ireland.
5. CRT government and expert guidelines (Additional file 3: Table DS6) (n = 20) [2,5,6,70-86]. These comprised eight sets of English government guidance, and 12 reports from UK voluntary sector campaigning or research organisations.

**Table 1:** **Comparison studies of different CRT models - study characteristics and outcomes**

Study Reference	MMAT score	Study characteristics	CRT models compared	Results
Allen (2009) [31]	n/a	Natural experiment with pre- and post-comparison; Buckinghamshire, UK	CRT team pre and post several organisational changes: “patient typing” system categorising service user needs introduced; greater use of leave from hospital to promote early discharge; referrals to CRT from other mental health services accepted without reassessment; structured screening tools introduced for acute assessments; closer links between CRT and day hospital introduced with single key worker system	Reduction in inpatient bed provision and greater service user satisfaction reported following changes. No statistical tests or numerical results reported
Doyle (1994) [32]	3	Natural experiment with parallel groups: 1 Team in Folkestone, UK 1 Team in Barnet, UK	Folkestone CRT with 9 am-5 pm opening hours	No clear difference between CRT models reported and no statistical tests reported. Over the follow-up period
			Barnet CRT with 24 hour opening	
				9% of the 9-5 (Folkestone) CRT service users were admitted to hospital vs 5% Barnet
Happell (2009) [33]	3	Natural experiment with pre- and post-comparison of parallel groups; Melbourne, Australia	Control Group: Day after initial assessment, full assessment given by *trainee psychiatrist*, care management plan formulated.	Mean HoNOS scores not significantly different between the groups at baseline – no p-values reported
			Treatment Group: Day after initial assessment, full assessment given by *nurse practitioner*, who took role of trainee psychiatrist (After 7 days service users in nurse-initiated care group reverted to treatment as usual)	HoNoS scores for both groups significantly improved with treatment (difference for control group: t = 7.90, df = 51, p < .001; difference for treatment group: t = 6.90, df = 50, p < .001) No information given as to whether there was a significant difference between group HoNOS scores after treatment.
				Service user and carer satisfaction scores were reported as not significantly different between groups – no p-values given
Harrison (2011) [34]	3	Natural experiment with pre- and post-comparison of a single CRT; Manchester, UK	• In 2005 (‘pre’), referrals were only taken from secondary services.	• 301 people treated in a six-month period in 2008/09, 128 in a comparable period in 2005
			• In 2008-2009 (‘post’), referral routes extended to primary care.	
				• Mean duration of contact in 2008/09 – 24 days, in 2005 – 69 days
				• 39% in 08/09 already known to services and in receipt of Care Plan Assessment (CPA), 70% in 05 (P < 0.005)
				• Increase in proportion treated for less severe illnesses (less severe depression and other diagnoses) in 2008/09 compared with 2005 (increase from 25 to 50%, P < 0.0001)
				• Fewer treated with severe mental illness (schizophrenia and related disorders, bipolar disorder and psychotic depression); 50% in 08/09, 75% in 2005, P < 0.0001
Reding (1995) [35]	4	Retrospective natural experiment with pre- and post-comparison; Kalamazoo County, Michigan, USA	• Comparison of before and after the introduction of a psychiatrist to the team	There were significantly fewer state hospital admissions in the team with a psychiatrist (p < 0.001). (The decrease in state hospital admissions was not offset by a corresponding increase in admissions to the local private psychiatric hospital.)

The overall mean quality score for included studies (not including government and expert guidelines) was 2.96 (moderately high quality) on the MMAT scale [29], with a standard deviation (SD) of 1.07. The breakdown of scores differed between types of study as follows: studies comparing two or more CRTs obtained a mean score of 3.25 (SD = 0.5); studies comparing a CRT to treatment as usual (TAU) or another service obtained 3.33 (SD = 0.72); the mean score of national surveys was 3.75 (SD = 0.5); and stakeholder interviews and surveys had a mean score of 2.61 (SD = 1.12). The results of one of the studies [44] are reported only briefly in a book chapter and consequently scored an MMAT rating of zero. The MMAT scores are reported in Additional file 4: Tables DS7-10.

### Results of studies

#### Quantitative comparison studies of two CRT models

Characteristics and results of comparison studies of two CRT models are summarised in Table 1. Of the five quantitative studies comparing two different CRT models, one [35] reported an association between the presence of a psychiatrist within the CRT and reduced hospital admissions (admissions reduced 40% (from 105 to 62), p < .0005). Harrison and colleagues [34] reported an association between extending direct referrals to primary care and a reduction in the proportion of CRT service users with severe and enduring mental illness and the mean duration of CRT care episodes (after introduction of primary care referrals, the percentage of people treated who had complex care needs reduced from 70% to 39%, p < 0.001). However the impact of this change in referral criteria on client or service outcomes was not evaluated. Three studies found no clear or significant difference between outcomes of the different CRT models regarding: organisational changes within the same team [31]; team opening times (9 am-5 pm versus 24-hours) [32]; and assessments by trainee psychiatrist versus by nurse practitioner [33].

#### Quantitative comparison studies of CRTs versus TAU

Full results from studies comparing CRTs with TAU (standard care not including a CRT) are provided in Additional file 5: Table DS11. Of the 16 studies, 13 used hospital admission as an outcome. Nine out of these 13 studies found reduced hospital admissions with CRT care. Four out of 12 studies looking at bed days found reduced bed days with CRT care; a further study found a significantly lower number of bed days in CRT group than for standard care at six weeks but not maintained at six months; and another reported reduced bed days but with no significance value. Of the five studies measuring service user satisfaction, two did not find greater satisfaction for service users using CRT services, whilst three found significantly higher satisfaction rates for CRT service users than those accessing treatment as usual or another service.

Additional file 5: Table DS12 provides full details of the characteristics of services in studies comparing CRTs with TAU. Data extraction regarding CRT characteristics remained incomplete, despite efforts to contact authors in order to fill in gaps in information. From the available data, at least 16 of the 20 CRTs provided medical cover including a psychiatrist within the team, 14 functioned with a gatekeeping role, 13 ran a 24 hour service, 13 were multi-disciplinary, nine facilitated early discharge and five had staff ratios of at least 14 per 150,000. Table 2 below summarises study outcomes and the characteristics of services in studies with positive results and those without.

**Table 2:** **CRTs versus Other services: Study outcomes and relationship to CRT characteristics**

Outcome	Results	Studies(MMAT score)	24 hour service	Gatekeeping role reported and implemented	Staffing (>14 staff per 150,000 population)	Medical cover within the CRT team	Multidisciplinary	Early discharge service
Inpatient admissions(admission at time of crisis)	Superior outcomes for CRT (n = 10)	Adesanya 2005 (4) [36]	7 papers = Yes (24 hour service was present)	8 = Yes (had gatekeeping role)	4 = No (staffing not adequate)	8 = Yes (had medical cover)	8 = Yes (multidisciplinary)	5 = Yes (had early discharge service)
		Barker 2011 (2) [12]						
		Dibben 2008 (3) [39]		2 = No (no gatekeeping role)	6 = Not reported	1 = No (no medical cover)	1 = No (not multidisciplinary)	
		Guo 2001 (4) [41]	2 papers = No (24 hour service was not present)					
		Hugo 2002 (4) [42]						1 = No (no early discharge service)
		Jethwa 2007 (3) [43]				1 = Not reported		
		Johnson 2005a (3) [8]	1 = Characteristic not reported					
		Johnson 2005b (3) [9]					1 = Not reported	
		Keown 2007 (4) [45]						4 = Not reported
		Piggott 1993(4) [47]						
	No significant difference between groups (n = 3)	Forbes 2010 (3) [40]	1 = Yes (24 hour service)	2 = Yes (had gatekeeping role)	2 = Yes (staffing adequate)	2 = Yes (had medical cover)	1 = Yes (multidisciplinary)	1 = Yes (had early discharge service)
		Kolbjornsrud 2009 (4) [46]						
			2 = No (no 24-hour service)	1 = No (no gatekeeping role)	1 = Not reported	1 = Not reported	1 = No (not multidisciplinary)	
		Tyrer 2010 (2) [13]						
								1 = No (no early discharge service)
							1 = Not reported	
								1 = Not reported
Inpatient bed days	Superior outcomes for CRT (n = 6)	Barker 2011 (2) [12]	5 = Yes (24 hour service)	5 = Yes (had gatekeeping role)	1 = Yes (staffing adequate)	5 = Yes (had medical cover)	4 = Yes (multidisciplinary)	4 = Yes (had early discharge service)
		Dean 1993 (3) [38]						
		Johnson 2005a* (3) [8]	1 = Not reported	1 = Not reported	2 = No (staffing not adequate)	1 = Not reported	2 = Not reported	
		Johnson 2005b (3) [9]						2 = Not reported
		Johnson 2008 (0) [44]			3 = Not reported			
		Piggott 1993 (4) [47]						
	No significant difference between groups (n = 6)	Adesanya 2008 (4) [36]	4 = Yes (24 hour service)	5 = Yes (had gatekeeping role)	1 = Yes (staffing adequate)	3 = Yes (had medical cover)	4 = Yes (multidisciplinary)	2 = Yes (had early discharge service)
		Bechdolf 2011 (4) [37]						
		Dibben 2008 (3) [39]	2 = No (no 24-hour service)	1 = Not reported	1 = No (staffing not adequate)	3 = Not reported	2 = Not reported	
		Forbes 2010 (3) [40]						
					4 = Not reported			1 = No (no early discharge service)
		Keown 2007 (4) [45]						
		Tyrer 2010 (2) [13]						
								3 = not reported
Service user satisfaction	Superior outcomes for CRT (n = 3)	Johnson 2005a (3) [8]	3 = Yes (24 hour service)	2 = Yes (had gatekeeping role)	1 = Yes (staffing adequate)	2 = Yes (had medical cover)	2 = Yes (multidisciplinary)	2 = Yes (had early discharge service)
		Johnson 2005b(3) [9]						
		Johnson 2008 (0) [44]		1 = Not reported	2 = Not reported	1 = Not reported	1 = Not reported	1 = Not reported
	No significant difference (n = 2)	Dibben 2008 (3) [39]	1 = Yes (24 hour service)	2 = Yes (had gatekeeping role)	2 = Not reported	1 = No (no medical cover)	1 = Yes (multidisciplinary)	1 = No (no early discharge service)
		Tyrer 2010 (2)	1 = No (no 24-hour service)			1 = Not reported	1 = Not reported	1 = Not reported

*Johnson [8] was included as one of the studies reporting superior outcomes for CRTs for bed use: it found reduced bed use in CRT group at 6 weeks follow up, though not at 6 month follow-up.

There was no obvious difference in study quality between studies reporting positive results and those not finding significant advantages to CRT care (median MMAT score of 3 for both). With the exception of staffing levels, where there was considerable missing data, in most studies the CRT was adhering to key elements of the original CRT model [2]. However, in the absence of any quantitative data synthesis, significant differences between effective and ineffective CRT services cannot be identified. No characteristic was consistently associated exclusively with better outcome or with no effect. The range of outcomes assessed was limited, with for example symptoms and quality of life not measured in most studies.

#### National/regional CRT surveys

An English CRT survey [7] reported that CRTs which offered a 24 hour service were more effective in reducing hospital admissions than those only operating reduced hours (83% of primary care trusts with a CRT with 24-hours service showed a fall in total admissions, compared with 60% of trusts with no team and 74% of trusts with a CRT without a 24-hour service). However, a secondary analysis of this data [14] casts some doubt on whether CRTs were effective in reducing admissions and suggested that it was not possible to isolate the impact of CRTs independent of co-occurring local reductions in inpatient beds. A Norwegian CRT survey [19] provided inferential evidence in support of CRTs operating with extended opening hours and accepting self-referrals if they sought to focus on working with acutely unwell people (CRTs with extended opening hours accepted more severely ill service users (HoNOS score p < 0.001) than those operating office hours only). CRTs with longer opening hours accepted more severely unwell service users, while accepted service users who had self-referred were as severely unwell as those referred by health professionals (see Additional file 3: Table DS4). A study investigating the same cohort reported that a team focus on out-of-office contact (unstandardized multivariate regression coefficients 2.502, p = 0.016) and longer treatment times (unstandardized multivariate regression coefficients 0.068, p < 0.001) were predictors of favourable outcomes of crises [48].

#### Qualitative studies of stakeholders views on CRTs

Findings from surveys, interviews, focus groups and quantitative questionnaires are fully reported and displayed thematically in the table in Additional file 5: Table DS13. The characteristics most frequently identified by service users, carers and staff as important elements influencing CRT service quality are summarised in Table 3.

**Table 3:** **Most commonly reported themes from qualitative studies of CRT stakeholders’ views**

CRT characteristic recommended by stakeholders	Number of studies where this theme was reported (n)
Good communication and integration with other mental health services	n = 14
Provision of treatment at home	n = 11
Limiting the number of different staff visiting a service user	n = 10
Adequate staffing, including out of hours	n = 9
Good staff record keeping and information sharing	n = 8
Staff with time and willingness to “just listen” to service users	n = 8
Rapid CRT response and availability of treatment during a crisis	n = 8
Clear, inclusive eligibility criteria	n = 8
CRTs provide a clear bridge to longer term interventions and care	n = 8

#### Government and expert guidelines

Additional file 5: Table DS14 reports the themes found in guidance and recommendations for CRTs in England. Documents included government and expert guidance publications, some of which are based on stakeholder groups such as NICE guidelines and reviews. Key elements of a CRT model which were specified in the original government mandatory guidelines regarding CRTs in England [2] were referenced in the documents, including: 24-hour, seven-day-a-week service; gatekeeping role; multidisciplinary teams; length of treatment; and staff numbers. The most common recommendations from CRT guidance are summarised in Table 4.

**Table 4:** **Most common recommendations for CRTs from English government and non-statutory organisations**

CRT characteristic recommended by guidance	Number of sources recommending this characteristic (n)
CRTs offer a 24-hour, 7 day a week service	n = 10
CRTs include a psychiatrist/medical cover	n = 10
High quality staff training in crisis home treatment	n = 6
CRTs have a multidisciplinary staff team	n = 6
CRTs act as gatekeepers for hospital admissions	n = 6
CRTs provide intensive, supportive interventions	n = 6
CRTs allocate a named worker for each service user	n = 6
Discharge from the CRT involves relapse prevention planning	n = 6
CRTs remain involved until a crisis has resolved	n = 6
CRTs undertake high quality auditing and service monitoring	n = 6

Other less frequent recommendations related to themes of medication management within the CRT; service user age and presentation to be served by the CRT; central location of the CRT; rapid assessment and acceptance of referrals from multiple sources; the role of medication, assessment, skilled staff, a team approach, short-term duration, location in the home and suitable referral to other services; content and process of care including risk, training and supervision, service user and carer involvement in care, and working with other services; risk policies and shared responsibilities; the extent of training and supervision of CRT staff; evaluation and monitoring to be carried out by the CRT; and joint working with other services. There was a high level of overlap and congruence between recommendations reported by statutory and by non-statutory organisations.

## Discussion

### Main findings

The review included 49 studies related to the implementation of CRTs in adult mental health settings, and 20 documents reporting government or expert guidance. Limited evidence from quantitative studies suggested that CRTs can reduce hospital admissions and increase service user satisfaction in some circumstances, but there is no robust evidence on which to base conclusions about the specific characteristics of CRTs which influence their effectiveness. There is some empirical support for the inclusion of a psychiatrist within the CRT [35], and provision of a 24-hour service rather than reduced operating hours [7,19].

Qualitative studies and CRT guidelines provided more specific suggestions for how to optimise CRT services, though they were generally based mainly on experience, personal views, and consensus processes. Stakeholders valued accessibility, continuity of care, provision of time to talk, practical help, and treatment at home. Guidelines emphasized that CRTs should provide a multi-disciplinary, 24-hour, short-term service to people experiencing a mental health crisis; and fulfil a gatekeeping role, controlling access to local inpatient beds. The importance of adequate staffing levels and staff skills was also stressed.

This review suggests there is substantial variation in how CRTs operate – such as staffing levels and whether or not teams had a fully implemented gatekeeping role – which may help explain variation in service outcomes. However, the original model for CRTs in England, specified in the Department of Health’s Policy Implementation Guide [2], appears to remain broadly supported by stakeholders, guidelines, and the little evidence available from quantitative studies. Moreover, the views of different stakeholder groups do not conflict, although they reflect differences of emphasis: guidelines and professional stakeholders focus on team resources and organisation, while service users and carers prioritise the content and experience of care. This suggests some consensus from which to develop a more highly specified and defined model of CRT care than is currently available, although it is currently a model with limited empirical basis.

### Strengths and limitations

This review used a systematic search strategy to collate all available types of evidence regarding the critical components of CRT services. Efforts were made to ensure we retrieved all relevant research studies: we supplemented a multi-database search with hand-searching of reference lists, and contacted authors about conference abstracts. Due to resource limitations however, the web-based search for government and expert guidelines was limited to England.

Three further limitations of the review should be acknowledged:

Firstly, the wide variation among studies in study design and quality and regarding CRT implementation, outcomes measured, and setting and study populations – together with substantial missing data regarding the characteristics of CRT teams – meant that we could not carry out quantitative synthesis of results from quantitative studies. This limited the direct comparison of the effectiveness of CRTs in different studies. An example of such a synthesis is the meta-regression conducted by Burns and colleagues [87], which usefully identified components of intensive case management services associated with reductions in inpatient bed use.

Secondly, the quality assessment measure used in this review was relatively crude. The retrieval of papers using a mixture of methods meant that the MMAT [29] numerical scale of quality assessment was the most appropriate available means of synthesising quality of evidence. In order to counterbalance subjective elements of scoring, assessment was carried out by two authors and disagreements resolved by a third. However, there are limitations inherent in conducting an assessment of the risk of bias in retrieved papers through the use of a scale that ‘numerically summarise[s] multiple components into a single number’ and therefore reduces evidence of quality to pre-specified categories [26,88]. A further limitation is that the MMAT treats different methodologies as equivalent, for example there is no weighting for RCTs compared to natural experiments. We used the MMAT because, to our knowledge, it is the best available single measure for assessing quality of studies with the range of different methodologies included in our review.

Thirdly, the inclusion of studies with lower quality scores may compromise the strength of conclusions. No formal assessment of selective outcome reporting or publication bias was undertaken; however, the high number of papers in this review with non-significant results suggests that publication bias might not be a problem. We decided to include all studies, regardless of quality, in order to gauge the current evidence base for the implementation of CRTs in adult mental health settings. Conclusions were drawn with reference to the variability of quality scores of the included studies.

### Implications for research

Despite identifying over 20 CRT outcomes studies, this review identified few empirically-based critical components of CRT services. Many of the studies were not designed to assess specific service characteristics, for example some were local service evaluations with limited statistical analysis that did not allow confounding to be taken fully into account. Future trials of CRTs should describe the CRT service and comparison services fully, as recommended by CONSORT Guidelines [89]. A priority for future CRT research is the development of a highly specified CRT model and means to assess adherence to this model and its relationship to outcomes.

CRTs are highly complex and contain a large number of varying components, which creates a methodological challenge to exploring the relationship between service characteristics and outcomes. It might not, therefore, be reasonable or feasible to carry out randomised controlled trials testing the effect of varying individual components for every element of CRT delivery. A potential alternative would be to study service characteristics and interventions delivered across large numbers of teams, investigating associations with outcomes at individual level using multilevel modelling. Contextual variables such as local service organisation and area geography could also be included. A fidelity approach (using a ‘tool to measure the implementation of an evidence-based practice’ [90]) offers a framework involving the development of a scale that captures the characteristics that stakeholders believe may be important. This approach has already been developed for other complex mental health interventions such as Assertive Community Treatment [91] and supported employment [92].

The findings from this review regarding stakeholders’ views and priorities for CRT service organisation and delivery can generate numerous hypotheses which could be tested in future research. Further evidence is required regarding the influence on outcomes of CRT characteristics such as: 24 hour opening, an exclusive gatekeeping role, named workers in teams, and a multi-disciplinary staff team. Further evidence regarding the content of care – i.e. how specific interventions such as brief psychological therapies or peer support programmes delivered by CRTs affect outcomes – would also contribute to knowledge on how to optimise CRT services.

It is notable from our review that service use (hospital admission or inpatient bed-days) is by far the most commonly studied outcome, with user-satisfaction with services a clear second. There is little evidence for the impact of CRTs on clinical outcomes such as symptom reduction or relapse, or on carers’ experience. These may require exploration in future studies, although a previous UK randomised trial found no impact on other outcomes from short term interventions that CRTs provide [9]. Topics for further scrutiny include the impact of CRT characteristics on compulsory admissions (most studies suggest it is primarily voluntary admissions that are affected) and readmissions to acute care. Whether CRTs are equally effective for all client groups also remains unclear.

### Implications for policy and practice

While not conclusive, there is some empirical basis for recommending that CRTs should provide extended opening hours and include a psychiatrist within the CRT team. Good consensus across qualitative research also suggests CRT managers should prioritise ensuring staff have time to listen to service users’ concerns and not be exclusively task-focused, and should also be able to provide a range of support including help with practical problems. Managers should also seek means to promote continuity and limit the number of different staff a service user sees during an episode of CRT support: one way to achieve this would be to provide each service user with a named worker. The CRT model outlined in government guidance when CRTs were originally mandated in England [2] remains generally supported by the limited available evidence.

In the absence of clearer evidence about the crucial components of CRT services, the impact of service changes in CRTs may be hard to anticipate for service planners and managers. Service developments within CRTs should, wherever possible, therefore be accompanied by rigorous service evaluation to assess their effects and add to knowledge about how to optimise this important aspect of mental health crisis care.

## Conclusions

Overall, the present findings provide considerable evidence about stakeholders’ priorities for CRTs, which are broadly congruent across stakeholder groups. However, our review allows few confident conclusions about the critical components for effectiveness of CRT services, due to the paucity of empirical evidence in the literature. Further research is required to determine elements of best practice that result in effective CRT service provision, including tools to evaluate adherence to a model of good practice.

## Additional files

Additional file 1:
**Microsoft Word document: PRISMA checklist.**

Additional file 2: **Microsoft Word document.** Search terms. **Table DS1.** CRT implementation Review - search terms for electronic database search. **Table DS2.** CRT implementation review - web resources searched for government and expert organisations’ guidelines.
Additional file 3: **Microsoft Word document.** Data extraction tables. **Table DS3.** CRT implementation review - study characteristics: CRTs vs TAU **Table DS4.** CRT implementation review - study characteristics and findings for CRT surveys. **Table DS5.** CRT implementation review - study characteristics from CRT stakeholder interviews, questionnaires and surveys.
Additional file 4: **Microsoft Word document.** MMAT scoring for studies included in this review. **Table DS7.** CRT implementation review - MMAT scores for studies comparing two CRTs. **Table DS8.** CRT implementation review - MMAT scores for studies comparing a CRT with TAU. **Table DS9.** CRT implementation review - MMAT scores for CRT surveys. **Table DS10.** CRT implementation review - MMAT scores for stakeholder interviews, surveys and questionnaires.
Additional file 5: **Microsoft Word document.** Tables of results and CRT characteristics. **Table DS11.** CRT implementation review - team characteristics and study outcomes for CRTs compared to TAU. **Table DS12.** CRT implementation review - CRTs versus other services: Further details of service characteristics. **Table DS13.** CRT implementation review - Stakeholders’ views on CRTs: themes and recommendations from included. **Table DS14**: CRT implementation review - recommendations from included government and expert guidelines.

## Abbreviations

CRTs: Crisis Resolution Teams
MMAT: Mixed methods appraisal tool
RCT: Randomised controlled trial
