# Supplementary material for: Implementation of the Crisis Resolution Team model in adult mental health settings: a systematic review
Source: BMC Psychiatry. 2015 Apr 8;15:74. doi: 10.1186/s12888-015-0441-x (PMC4405828; doi:10.1186/s12888-015-0441-x)
Supplement: Additional file 1: — Microsoft Word document: PRISMA checklist. [file 12888_2015_441_MOESM1_ESM.doc]

| **Section/topic** | **#** | **Checklist item** | **Reported on page #** |
| --- | --- | --- | --- |
| **TITLE** | | |  |
| Title | 1 | **Implementation of the Crisis Resolution Team model in adult mental health settings: A systematic review** | 1 |
| **ABSTRACT** | | |  |
| Structured summary | 2 | **Background**  Crisis Resolution Teams (CRTs) aim to offer an alternative to hospital admission during mental health crises, providing rapid assessment, home treatment, and the facilitation of early discharge from hospital. CRTs were implemented nationally in England following the NHS Plan of 2000. Single centre studies suggest CRTs can reduce hospital admissions and increase service users’ satisfaction: however, there is also evidence that both the way the model is implemented and outcomes vary considerably. Evidence on crucial characteristics of effective CRTs is needed to allow team functioning to be optimised. Our aim in this review was to establish what evidence, if any, is available regarding the characteristics of effective and acceptable CRTs.  **Methods**  A systematic review was conducted. MEDLINE, Embase, PsycINFO, CINAHL and Web of Science were searched to November 2013. A further web-based search was conducted for government and expert guidelines on CRTs. We analysed studies separately as: comparing CRTs to Treatment as Usual; comparing two or more CRT models; national or regional surveys of CRT services; qualitative studies of stakeholders’ views regarding best practice in CRTs; and guidelines from government and expert organisations regarding CRT service delivery. Quality assessment and narrative synthesis were conducted. Statistical meta-analysis was not feasible due to the variety of design of retrieved studies.  **Results**  Sixty-nine studies were included. Studies varied in quality and in the composition and activities of the clinical services studied. Quantitative studies suggested that longer opening hours and the presence of a psychiatrist in the team may increase CRTs’ ability to prevent hospital admissions. Stakeholders emphasised communication and integration with other local mental health services; provision of treatment in the home; and limiting the number of different staff members visiting a service user. Existing guidelines prioritised 24-hour, seven-day-a-week CRT service provision (including psychiatrist and medical prescriber); and high quality of staff training.  **Conclusions**  We cannot draw confident conclusions about the critical components of CRTs from available quantitative evidence. Clearer definition of the CRT model is required, informed by stakeholders’ views and guidelines. Future studies examining the relationship of overall CRT model fidelity to outcomes, or evaluating the impact of key aspects of the CRT model, are desirable.  **Trial registration:** Prospero CRD42013006415 | 2 |
| **INTRODUCTION** | | |  |
| Rationale | 3 | While trials suggest that Crisis Resolution Teams can be effective in reducing hospital admissions and increasing service users’ satisfaction with acute care, little is known about critical ingredients of CRT services and how the CRT model may be implemented most effectively. | 3 |
| Objectives | 4 | This review aims to systematically review randomised and non-randomised comparison studies and national surveys of CRT services, qualitative studies of CRT stakeholders’ views, and national and expert guidelines relating to the implementation of CRTs in England. We aim to investigate:  i. What characteristics of CRTs are associated with positive outcomes in empirical evaluations of CRT services?  ii. What do service users, carers and staff identify in qualitative studies and surveys and quantitative questionnaires as important elements influencing CRT service quality?  iii. What recommendations do government agencies and non-statutory organisations and experts make regarding CRT service delivery and organisation? | 4 |
| **METHODS** | | |  |
| Protocol and registration | 5 | Review protocol is registered with: PROSPERO International prospective register of systematic reviews  Registration number: Prospero CRD42013006415  [http://www.crd.york.ac.uk/prospero/display_record.asp?ID=CRD42013006415#.UqnXyuLDUko](http://www.crd.york.ac.uk/prospero/display_record.asp?ID=CRD42013006415#.UqnXyuLDUko) | 4 |
| Eligibility criteria | 6 | **Inclusion criteria**  ***Services***  We included studies of CRTs that offer intensive home treatment for a brief period (typically a month or less on average) to adults with acute mental health problems who would otherwise be admitted to hospital. We included specialist services established for crisis care and integrated services with a clear crisis function. For quantitative studies, comparison treatment as usual (TAU) groups were specialist mental health services that provide multi-disciplinary community-based care (such as UK Community Mental Health Teams).  We excluded studies of intensive home treatment services which offered on-going rather than brief care (such as Assertive Community Treatment teams). In order to assess the impact of CRTs in a contemporary mental health system involving secondary care community mental health teams, we also excluded studies comparing CRT services to treatment as usual where the latter involved only inpatient care or outpatient appointments with a psychiatrist.  ***Participants***  At the participant level, the inclusion criterion was that CRTs serve adults with acute mental health problems who would otherwise be admitted to hospital. Studies including older age adults were included if the participants had a functional mental illness rather than an organic mental disorder.  Studies primarily including participants under the age of 16 were excluded.  ***Types of study***  The following types of study were included:  1. Quantitative studies of any type comparing outcomes between two or more CRTs with different characteristics or service content  2. Quantitative studies of any type comparing a CRT service with another type of service or treatment as usual (in order to explore differences in CRT characteristics between studies where the CRT is found to have an association with improved outcomes and studies where there was no effect)  3. National or regional level surveys of CRTs which report associations between service characteristics and outcomes  4. Qualitative interviews, focus groups or surveys (some also including quantitative questionnaires) of stakeholders’ views (service users, carers and staff) regarding elements of good CRT services  5. Published guidelines from statutory agencies or non-statutory organisations with responsibility for policy and health services in England, which provided recommendations regarding CRT service delivery and organisation, often based on the views of an expert panel or a panel containing experts and stakeholder group representatives  In anticipation of few randomised trials being found, studies in categories 1) and 2) were not restricted by methodology: randomised controlled trials and also natural experiments with pre- and post- comparisons and natural experiments with parallel groups were eligible for inclusion.  Studies written in languages other than English were not excluded. Studies conducted up to the time of the last search were included, and there was no time limit specified. | 5-6 |
| Information sources | 7 | An electronic database search using MEDLINE, Embase, PsycINFO, CINAHL and Web of Science was conducted (last search conducted in November 2013).  Guidelines were searched from UK government sources: online publications archives from the Department of Health, the National Audit Office and the National Mental Health Development Unit; and from non-statutory organisations: the Kings Fund, the Centre for Mental Health, Rethink, MIND, the Royal College of Psychiatrists. | 6 |
| Search | 8 | MEDLINE search (noi restrictions using limit functions)  1. “Mental Disorders” OR “Mental Health Services” [MeSH terms]  2. “crisis intervention” or “crisis resolution” or “crisis assessment” or “home treatment” [in abstract] OR “Crisis Resolution” [MeSH term]  3. 1 AND 2 | 6 |
| Study selection | 9 | The title and abstract of all retrieved studies were scanned independently by two reviewers (AC, BLE, CF, BP or CW). The full text of potentially eligible papers was retrieved and decisions about inclusion made by two reviewers (AC, BLE, CF, BP or CW). We screened the reference lists of key papers. Any disagreement regarding inclusion was resolved through discussion or, where necessary, with reference to another reviewer (SJ). | 6 |
| Data collection process | 10 | A data extraction form was used to code and record relevant data from each included study. Data extraction was carried out by a member of the review team (BLE, CF, LM, BP, CW or CGZ) and checked by another member of the team; with discrepancies resolved in consultation with another reviewer (SJ). Information was extracted from included studies on: study characteristics, results and CR service characteristics  We contacted authors to ask for any of this information not available from published papers. | 6 |
| Data items | 11 | 1. Study characteristics: type of study; setting; participant numbers and characteristics (for quantitative studies); duration of study and outcomes assessed 2. Results: outcomes and significant findings from quantitative studies; themes and recommendations from stakeholder interviews and guidelines 3. CRT service characteristics: for quantitative studies comparing two CRT service models, we reported the differences between services being studied; for studies of CRTs versus standard care, we reported characteristics of CRTs identified in statutory guidance for England [2] including 24 hour service, gatekeeping function staffing levels, multi-disciplinary team, medical staffing in team, duration of care and early discharge function to support prompt discharge from hospital. | 6 |
| Risk of bias in individual studies | 12 | Quality was assessed using the Mixed Methods Appraisal Tool (MMAT). The tool is applicable to quantitative, qualitative and mixed methods primary studies. We did not exclude papers from the review on account of low quality scores, but quality scores were reported and considered in the narrative synthesis of the evidence. The MMAT quality scoring scale ranges from 0 (low quality) to 4 (high quality). The MMAT has been pilot tested for reliability in systematic reviews. Ratings are specific to particular methodologies, and are based on control of confounding factors; completeness of outcome data; minimisation of selection bias; representativeness of sample; appropriateness of measures; response and withdrawal rates; appropriateness of study design to answer the research questions; and consideration of limitations.  Quality assessment was conducted only at study level, not individual outcome level. All eligible studies were included in the review’s narrative synthesis, regardless of study quality. Quality of included studies was considered in reporting and discussing findings from the review. | 7 |
| Summary measures | 13 | N/A: no quantitative synthesis | - |
| Synthesis of results | 14 | Narrative synthesis | 7 |

Page 1 of 2

| **Section/topic** | **#** | **Checklist item** | **Reported on page #** |
| --- | --- | --- | --- |
| Risk of bias across studies | 15 | No formal assessment of selective outcome reporting or publication bias was undertaken; however, the high number of papers in this review with non-significant results is not suggestive of substantial publication bias. | 13 |
| Additional analyses | 16 | N/A: no additional analyses | - |
| **RESULTS** | | |  |
| Study selection | 17 | The Study Selection flow Diagram - Figure 1 - shows the selection and screening of papers to include in the review. After removing duplicates, the database search yielded 2749 studies. The web-based search for expert and government guidelines yielded 1650 papers/reports. After screening, 69 studies and documents were identified for inclusion in the review. | 8 |
| Study characteristics | 18 | The 69 papers included in the review comprised:   1. Comparisons of two CRT models (Table 1) (n=5) [31-35]: Natural experiments, three with pre-post comparisons. Studies were published between 1994 and 2011; three were set in the UK, one in USA and one in Australia. Outcomes assessed were admission rates, health status at discharge, and service user and carer satisfaction.   References to papers:  31. Allen A, Blaylock W, Mieczkowski S: Local implementation of the crisis model: the Buckinghamshire community acute service. Psychiatric Bulletin 2009, 33: 252-254.  32. Doyle H, Varian J: Crisis intervention in psychogeriatrics: A round-the-clock commitment? International Journal of Geriatric Psychiatry 1994, 9(1):65-72.  33. Happell B, Sundram S, Wortans J, Johnstone S, Ryan R, Lakshmana R:. Assessing nurse-initiated care in a mental health crisis assessment and treatment team in Australia. PSYCHIATR SERV 2009, 60(11):1527-1531.  34. Harrison J, Rajashankar S, Davidson S: From home treatment to crisis resolution: the impact of national targets. The Psychiatrist 2011, 35:89-91.  35. Reding GR, Raphelson M: Around-the-clock mobile psychiatric crisis intervention: another effective alternative to psychiatric hospitalization. Community Mental Health Journal 1995, 31(2):179-87.   1. CRTs versus standard care (Table DS3, Additional File 2) (n=16) [8,9,12,13,36-47]: Two studies were randomised controlled trials, three were non-randomised (naturalistic) two-group comparison studies; and 11 were naturalistic pre-post comparison studies. Two studies were Australian, one German, one American, and 12 British; studies were published between 1993 and 2011. Primary outcomes in the included studies were hospital admission rates and service user satisfaction ratings. Conclusions were drawn regarding the characteristics of CRTs in these studies in relation to their outcomes.   References to papers:  8. Johnson S, Nolan F, Hoult J, White IR, Bebbington P, Sandor A, McKenzie N, Patel SN, Pilling S: Outcomes of crises before and after introduction of a crisis resolution team. British Journal of Psychiatry 2005a, 187:68-75.  9. Johnson S, Nolan F, Pilling S, Sandor A, Hoult J, McKenzie N, White IR, Thompson M, Bebbington P:. Randomised controlled trial of acute mental health care by a crisis resolution team: the north Islington crisis study. BMJ 2005b, 331(7517):599.  12. Barker V, Taylor M, Kader I, Stewart K, Le Fevre P: Impact of crisis resolution and home treatment services on user experience and admission to psychiatric hospital. The Psychiatrist 2011, 35:106-110.  13. Tyrer P, Gordon F, Nourmand S, Lawrence M, Curran C, Southgate D, Oruganti B, Tyler M, Tottle S, North B, Kulinskaya E, Kaleekal JT, Morgan J: Controlled comparison of two crisis resolution and home treatment teams. The Psychiatrist Online 2010, 34:50-54.  36. Adesanya A: Impact of a crisis assessment and treatment service on admissions into an acute psychiatric unit. Australasian Psychiatry 2005, 13(2):135-139.  37. Bechdolf A, Skutta M, Horn A: Clinical Effectiveness of Home Treatment as Compared to Inpatient Treatment at the Alexianer Hospital Krefeld, Germany. Fortschritte der Neurologie Psychiatrie, 2011 79 (1):26-31.  38. Dean C, Phillips J, Gadd EM, Joseph M, England S: Comparison of community based service with hospital based service for people with acute, severe psychiatric illness. BMJ 1993, 307(6902):473-6.  39. Dibben C, Saeed H, Stagias K, Khandaker GM, Rubinsztein, JS: Crisis resolution and home treatment teams for older people with mental illness. Psychiatric Bulletin 2008, 32(7):268-270.  40. Forbes NF, Cash HT, Lawrie SM: Intensive home treatment, admission rates and use of mental health legislation. Psychiatrist 2010, 34(12):522-524.  41. Guo S, Biegel DE, Johnsen JA, Dyches H: Assessing the impact of community-based mobile crisis services on preventing hospitalization. Psychiatric Services 2001, 52(2):223-8.  42. Hugo M, Smout M, Bannister J: A comparison in hospitalization rates between a community-based mobile emergency service and a hospital-based emergency service. Australian & New Zealand Journal of Psychiatry 2002, 36(4):504-508.  43. Jethwa K, Galappathie N, Hewson P: Effects of a crisis resolution and home treatment team on in-patient admissions. Psychiatric Bulletin 2007, 31:170-172.  44. Johnson S, Bindman J: Recent research on crisis resolution teams: findings and limitations. In Crisis Resolution and Home Treatment in Mental Health. Edited by Johnson S, Needle J, Bindman J, Thornicroft G. Cambridge University Press; 2008: 51-65.  45.Keown P, Tacchi MJ, Niemiec S, Hughes J: Changes to mental healthcare for working age adults: impact of a crisis team and an assertive outreach team. Psychiatric Bulletin 2007, 31:288-292.  46. Kolbjornsrud OB, Larsen F, Elbert G, Ruud T: [Can psychiatric acute teams reduce acute admissions to psychiatric wards?]. [Norwegian]. Tidsskrift for Den Norske Laegeforening 2009 129(19):1991-1994.  47. Pigott HE, Trott L: Translating research into practice: the implementation of an in-home crisis intervention triage and treatment service in the private sector. American Journal of Medical Quality 1993, 8(3):138-144.   1. CRT national surveys (Table DS4, Additional File 2) (n=4) [7,14,19,48]: Two papers reported one UK national CRT survey; two papers reported one Norwegian national survey. The UK survey was first published in 2006; the Norwegian survey in 2011.   References to papers:  7. Glover G, Arts G, Babu KS: Crisis resolution/home treatment teams and psychiatric admission rates in England. British Journal of Psychiatry 2006, 189:441-445.  14. Jacobs R, Barrenho E: The Impact of Crisis Resolution and Home Treatment Teams on Psychiatric Admissions in England. Journal of Mental Health Policy and Economics 2011, 14:S13.  19. Hasselberg N, Grawe RW, Johnson S, Ruud T: An implementation study of the crisis resolution team model in Norway: are the crisis resolution teams fulfilling their role? BMC Health Services Research 2011a, 11:96.  48. Hasselberg N, Grawe RW, Johnson S, Ruud T: Treatment and outcomes of crisis resolution teams: A prospective multicentre study. BMC Psychiatry 2011b, 11:183.   1. CRT stakeholder qualitative interviews and quantitative surveys (Table DS5, Additional File 2) (n=24) [4,16,49-68]: Twelve studies included service users as participants, five included carers, and twelve included CRT staff. In 15 studies, individual interviews were conducted in person (seven semi-structured, two structured, six not reported); one involved focus groups and eight involved data collection via online surveys, postal questionnaire or phone interview. The studies included between 1 and 177 CRTs, and between 7 and 471 participants. 17 studies were set in the UK; two each in Australia and Norway; and one each in France, Canada and The Republic of Ireland.   References to papers  4. Onyett S, Linde K, Glover G, Loyd S, Bradley S, Middleton H: Implementation of crisis resolution/home treatment teams in England: national survey 2005 -2006. Psychiatric Bulletin 2008, 32:374-377.  16. Hopkins C, Niemiec S: Mental health crisis at home: service user perspectives on what helps and what hinders. Journal of Psychiatric and Mental Health Nursing 2007, 14(3):310-318.  49. ‘AMaze’, Shaw B, Stapleton V: Reality of Crisis. Becky Shaw: Nottinghamshire 2010.  50. Ampelas JF, Robin M, Caria A, Basbous I, Rakowski F, Mallat V, Zeltner L, Bronchard M, Mauriac F, Waddington A: Patient and their relatives' satisfaction regarding a home-based crisis intervention provided by a mobile crisis team. Encephale-Revue de Psychiatrie Clinique Biologique et Therapeutique 2005, 31 (2):127-141.  51. Armitage C, Lange F: Crisis resolution teams and the role of the service user development worker. Mental Health Practice 2006 9(6):15-17.  52. Borg M, Karlsson B, Kim HS: Double helix of research and practice-developing a practice model for crisis resolution and home treatment through participatory action research. Int J Qualitative Stud Health Well-being 2010, 5:4647.  53. Freeman J, Vidgen A, Davies-Edwards E: Staff experiences of working in crisis resolution and home treatment. Mental Health Review Journal 2011, 16(2):76-87.  54. Fulford M, Farhall J: Hospital versus home care for the acutely mentally ill? Preferences of caregivers who have experienced both forms of service. Australian & New Zealand Journal of Psychiatry 2001, 35(5):619-625.  55. Hannigan B: Mental Health services in transition: examining community crisis resolution and home treatment care. Final Report to the Research Capacity Building Collaboration Wales for Nursing and Allied Health Professionals (rcbcwales) 2010.  56. Karlsson B, Borg M, Kim HS: From good intentions to real life: introducing crisis resolution teams in Norway. Nursing Inquiry 2008, 15(3):206-15.  57. Khalifeh H, Murgatroyd C, Freeman M, Johnson S, Killaspy H: Home treatment as an alternative to hospital admission for mothers in a mental health crisis: A qualitative study. Psychiatric Services 2009, 60(5):634-639.  58. Lyons C, Hopley P, Burton CR, Horrocks J: Mental health crisis and respite services: Service user and carer aspirations. Journal of Psychiatric and Mental Health Nursing 2009 16(5):424-433.  59. McCauley M, Bergin A, Bannon H, McDonald B, Bedford D, Russell V: How do GPs experience home-based treatment for acute psychiatric disorders? Primary Care and Community Psychiatry 2005, 10(4):159-163.  60. Middleton H, Shaw R, Collier R, Purser A, Ferguson B: The dodo bird verdict and the elephant in the room: A service user-led investigation of crisis resolution and home treatment. Health Sociology Review 2011, 20(2):147-156.  61. Morgan S, Hunte K: One foot in the door. Mental Health Today 2008, :32-35.  62. Morton J: Crisis resolution: A service response to mental distress. Practice (09503153) 2009 21(3):143-158  63. NAO (National Audit Office): Helping people through mental health crisis: The role of Crisis Resolution and Home Treatment service. HC 5 Session 2007-2008, 2007.  64. Nelson T, Johnson S, Bebbington P: Satisfaction and burnout among staff of crisis resolution, assertive outreach and community mental health teams. A multicentre cross sectional survey. Social Psychiatry & Psychiatric Epidemiology 2009, 44(7):541-549.  65. Reynolds I, Jones JE, Berry DW, Hoult JE: A crisis team for the mentally ill: the effect on patients, relatives and admissions. Medical Journal of Australia 1990, 152(12):646-652.  66. Taylor S, Abbott S, Hardy S: The INFORM project: A service user-led research endeavour. Archived of Psychiatric Nursing 2012, 26(6):448-456.  67. Tobitt S, Kamboj S: Crisis resolution/home treatment team workers' understandings of the concept of crisis. Social Psychiatry and Psychiatric Epidemiology 2011, 46(8):671-683.  68. Wasylenki D, Gehrs M, Goering P, Toner B: A home-based program for the treatment of acute psychosis. COMMUNITY MENT HEALTH J 1997, 33(2):151-162.   1. CRT government and expert guidelines (Table DS6, Additional File 2) (n=20) [2,5-6,69-85]. These comprised eight sets of English government guidance, and 12 reports from UK voluntary sector campaigning or research organisations.   References to guidance documents:  2. Department of Health: The Mental Health Policy Implementation Guide. Department of Health 2001.  5. National Institute for Health and Care Excellence (NICE): Psychosis and schizophrenia in adults: Treatment and management [Online] [Retrieved on 2 July 2014 from http://www.nice.org.uk/Guidance/CG178] 2014.  6. Joint Commissioning Panel for Mental Health (JCPMH): Guidance for commissioners of acute care – Inpatient and crisis home treatment. [Online] [Retrieved on 2 July 2014 from http://www.jcpmh.info/good-services/acute-care-services/] 2013.  69. Department of Health: The NHS Performance Framework: Implementation Guide. NHS Finance, Performance & Operations Directorate 2011.  69. Weich S, Griffith L, Commander M, Bradby H, Sashidharan SP, Pemberton S, Jasani R, Bhui KS: Experiences of acute mental health care in an ethnically diverse inner city: Qualitative interview study. Social Psychiatry and Psychiatric Epidemiology 2012, 47:119-128.  70. Naylor C, Bell A: Mental Health and the Productivity Challenge. The King's Fund and The Sainsbury Centre for Mental Health 2001.  72. Royal College of Psychiatrists: Social Inclusion Scoping Group: Mental Health and Social Inclusion. Royal College of Psychiatrists 2009.  73. Controller and Auditor General: Helping people through mental health crisis: The role of Crisis Resolution and Home Treatment Services. National Audit Office 2007.  71. Davies P, Taylor J: Getting the medicines right 2: Medicines Management in Mental Health and Crisis Resolution and Home Treatment Teams. National Mental Health Development Unit 2010.  74. Department of Health: New Ways of Working for Everyone: A best practice guide. Department of Health 2007.  75. Crompton N, Daniel D: Guidance Statement on Fidelity and Best Practice for Crisis Services. Department of Health 2007.  76. McGlynn P Authors Bridgett C, Flowers M, Ford K, Hoult J, Lakhani N, McGlynn P, Woodbridge K (Eds): Crisis Resolution and Home Treatment: A practical guide. Sainsbury Centre for Mental Health 2006.  77. Bell A, Lindley P (eds.): Beyond the Water Towers: The unfinished revolution in mental health services 1985-2005. Sainsbury Centre for Mental Health 2005.  78. Chisholm A, Ford R: Transforming Mental Health Care: Assertive Outreach and Crisis Resolution in Practice. Sainsbury Centre for Mental Health 2004.  79. Rethink: Carers Under Pressure. Rethink 2003.  80. Sainsbury Centre for Mental Health: Setting up and Running Crisis Resolution Services. Sainsbury Centre for Mental Health 2001.  81. Workforce Action Team for NHS: Mental Health National Service Framework: Workforce Planning, Education, and Training. Workforce Action Team for NHS 2001.  82. Worthington A, Rooney P: The Triangle of Care. Carers included: a guide to best practice in acute mental health care. National Mental Health Development Unit 2010.  83. National Collaborating Centre for Mental Health (NCCMH): Service user experience in adult mental health. NICE guidance on improving the experience of care for people using adult NHS mental health services. British Psychological Society and The Royal College of Psychiatrists [Online] [Retrieved on 4 July 2014 from http://www.nice.org.uk/guidance/CG136] 2012.  84. Centre for Social Justice, Mental Health Working Group chaired by Samantha Callan: Completing the revolution. Transforming mental health and tackling poverty. Centre for Social Justice [Online] [Retrieved on 4 July 2014 from http://www.centreforsocialjustice.org.uk/UserStorage/pdf/Pdf%20reports/CompletingtheRevolution.pdf] 2011.  85. The Schizophrenia Commission: The Abandoned Illness. The Schizophrenia Commission [Online] [Retrieved on 4 July 2014 from http://www.rethink.org/media/514093/TSC_main_report_14_nov.pdf] 2012. | P8 (summary)  P16-22 (references)  Additional file 2 (study characteristics) |
| Risk of bias within studies | 19 | MMAT scores for included studies (range 0-4):  CRT versus CRT comparison studies  Allen 2009: n/a  Doyle 1994: 3  Happell 2009: 3  Harrison 2011: 3  Reding 1995: 4  CRT versus TAU studies  Adesanya 2005: 4  Barker 2011: 2  Bechdolf 2011: 4  Dean 1993: 3  Dibben 2008: 3  Forbes 2010: 3  Guo 2001: 4  Hugo 2002: 4  Jethwa 2007: 3  Johnson 2005a: 3  Johnson 2005b: 3  Johnson 2008: 0  Keown 2007: 4  Kolbjornsrud 2009: 4  Pigott 1993: 4  Tyrer 2010: 4  CRT national surveys  Glover 2006: 3  Hasselberg 2011a: 4  Jacobs 2011: 4  Hasselberg 2011b: 4  CRT Qualitative studies  Amaze 2010: 3  Ampelas 2005: 4  Armitage 2006: n/a  Borg 2010: 2  Freeman 2011: 3  Fulford 2010: 2  Hannigan 2010: 4  Hopkins 2007: 2  Karlsson 2008: 1  Khalifeh 2009: 2  Lyons 2009: 2  McCauley 2005: 2  Middleton 2011: 3  MIND 2011: n/a  Morgan 2008: 0  Morton 2009: 4  NAO 2007: n/a  Nelson 2009: 3  Onyett 2008: 3  Reynolds 1990: 3  Taylor 2012: 2  Tobitt 2011: 4  Wasylenki 1997: 1  Weich 2012: 4  No MMAT ratings were made for government and non-statutory guidance documents. | Additional file 3 |
| Results of individual studies | 20 | Quantitative comparison studies of two CRT models  Of the five quantitative studies comparing two different CRT models, one [35] reported an association between the presence of a psychiatrist within the CRT and reduced hospital admissions (admissions reduced 40% (from 105 to 62), p<.0005). Harrison and colleagues [34] reported an association between extending direct referrals to primary care and a reduction in the proportion of CRT service users with severe and enduring mental illness and the mean duration of CRT care episodes (after introduction of primary care referrals, the percentage of people treated who had complex care needs reduced from 70% to 39%, p<0.001). However the impact of this change in referral criteria on client or service outcomes was not evaluated. Three studies found no clear or significant difference between outcomes of the different CRT models regarding: organisational changes within the same team [31]; team opening times (9am-5pm versus 24-hours) [32]; and assessments by trainee psychiatrist versus by nurse practitioner [33].  CRT versus TAU studies  Of the 16 studies, 13 used hospital admission as an outcome. Nine out of these 13 studies found reduced hospital admissions with CRT care. Four out of 12 studies looking at bed days found reduced bed days with CRT care; a further study found a significantly lower number of bed days in CRT group than for standard care at six weeks but not maintained at six months; and another reported reduced bed days but with no significance value. Of the five studies measuring service user satisfaction, two did not find greater satisfaction for service users using CRT services, whilst three found significantly higher satisfaction rates for CRT service users than those accessing treatment as usual or another service.  Table DS12, Additional File 4 provides full details of the characteristics of services in studies comparing CRTs with TAU. Data extraction regarding CRT characteristics remained incomplete, despite efforts to contact authors in order to fill in gaps in information. From the available data, at least 16 of the 20 CRTs provided medical cover including a psychiatrist within the team, 14 functioned with a gatekeeping role, 13 ran a 24 hour service, 13 were multi-disciplinary, nine facilitated early discharge and five had staff ratios of at least 14 per 150,000.  CRT national surveys  An English CRT survey [7] reported that CRTs which offered a 24 hour service were more effective in reducing hospital admissions than those only operating reduced hours (83% of primary care trusts with a CRT with 24-hours service showed a fall in total admissions, compared with 60% of trusts with no team and 74% of trusts with a CRT without a 24-hour service). However, a secondary analysis of this data [14] casts some doubt on whether CRTs were effective in reducing admissions and suggested that it was not possible to isolate the impact of CRTs independent of co-occurring local reductions in inpatient beds. A Norwegian CRT survey [19] provided inferential evidence in support of CRTs operating with extended opening hours and accepting self-referrals if they sought to focus on working with acutely unwell people (CRTs with extended opening hours accepted more severely ill service users (HoNOS score p<0.001) than those operating office hours only). CRTs with longer opening hours accepted more severely unwell service users, while accepted service users who had self-referred were as severely unwell as those referred by health professionals (see Table DS4, Additional File 2). A study investigating the same cohort reported that a team focus on out-of-office contact (unstandardized multivariate regression coefficients 2.502 , p=0.016) and longer treatment times (unstandardized multivariate regression coefficients 0.068 , p<0.001) were predictors of favourable outcomes of crises [48].  CRT qualitative studies  The characteristics most frequently identified by service users, carers and staff as important elements influencing CRT service quality were:  • good communication and integration with other local mental health services (referenced in 14 out of the 24 documents)  • provision of treatment in the home where possible (11 references)  • limiting the number of different staff members visiting a service user (10 references)  Other commonly referenced themes were:  • CRT teams need adequate staffing to meet demands, especially out of hours (9 references)  • CRTs should ensure good record keeping and information-sharing between staff (8 references)  • CRTs should provide staff time for service users to have someone ‘just listen’ (8 references)  • CRTs should provide immediate treatment during crisis and with a prompt response (8 references)  • CRTs should provide clear and not-too-restrictive eligibility criteria (8 references)  • CRTs should provide a clear bridge between short- and long-term interventions (8 references)  Government and non-statutory guidelines  The recommendations most frequently made for CRTs by government and non-statutory organisations were for 24-hour, seven-day-a-week CRT service provision (including psychiatrist and medical prescriber) (referenced in 10 of the 20 documents); and high quality of staff training (in 6 documents). Other frequent recommendations were for the CRT to be a multidisciplinary team; the CRT to act as a gatekeeper for hospital admissions; the provision of intensive and supportive intervention from a named worker (service users are allocated a particular CRT staff member); relapse prevention to be dealt with in discharge planning; and CRT to be involved until the crisis is resolved; and good quality auditing and monitoring (all referenced in 6 of the 20 documents). Other less frequent recommendations related to themes of medication management within the CRT; service user age and presentation to be served by the CRT; central location of the CRT; rapid assessment and acceptance of referrals from multiple sources; the role of medication, assessment, skilled staff, a team approach, short-term duration, location in the home and suitable referral to other services; content and process of care including risk, training and supervision, service user and carer involvement in care, and working with other services; risk policies and shared responsibilities; the extent of training and supervision of CRT staff; evaluation and monitoring to be carried out by the CRT; and joint working with other services. There was a high level of overlap and congruence between recommendations reported by statutory and by non-statutory organisations. | P 9-11 |
| Synthesis of results | 21 | No quantitative data synthesis/meta-analysis undertaken | - |
| Risk of bias across studies | 22 | The overall mean quality score for included studies (not including government and expert guidelines) was 2.96 (moderately high quality) on the MMAT scale [29], with a standard deviation (SD) of 1.07. The breakdown of scores differed between types of study as follows: studies comparing two or more CRTs obtained a mean score of 3.25 (SD = 0.5); studies comparing a CRT to treatment as usual (TAU) or another service obtained 3.33 (SD = 0.72); the mean score of national surveys was 3.75 (SD = 0.5); and stakeholder interviews and surveys had a mean score of 2.61 (SD = 1.12). | P 8-9 |
| Additional analysis | 23 | N/A | - |
| **DISCUSSION** | | |  |
| Summary of evidence | 24 | The review included 49 studies related to the implementation of CRTs in adult mental health settings, and 20 documents reporting government or expert guidance. Limited evidence from quantitative studies suggested that CRTs can reduce hospital admissions and increase service user satisfaction in some circumstances, but there is no robust evidence on which to base conclusions about the specific characteristics of CRTs which influence their effectiveness. There is some empirical support for the inclusion of a psychiatrist within the CRT [35], and provision of a 24-hour service rather than reduced operating hours [7,19].  Qualitative studies and CRT guidelines provided more specific suggestions for how to optimise CRT services, though they were generally based mainly on experience, personal views, and consensus processes. Stakeholders valued accessibility, continuity of care, provision of time to talk, practical help, and treatment at home. Guidelines emphasized that CRTs should provide a multi-disciplinary, 24-hour, short-term service to people experiencing a mental health crisis; and fulfil a gatekeeping role, controlling access to local inpatient beds. The importance of adequate staffing levels and staff skills was also stressed.  This review suggests there is substantial variation in how CRTs operate – such as staffing levels and whether or not teams had a fully implemented gatekeeping role – which may help explain variation in service outcomes. However, the original model for CRTs in England, specified in the Department of Health’s Policy Implementation Guide [2], appears to remain broadly supported by stakeholders, guidelines, and the little evidence available from quantitative studies. Moreover, the views of different stakeholder groups do not conflict, although they reflect differences of emphasis: guidelines and professional stakeholders focus on team resources and organisation, while service users and carers prioritise the content and experience of care. This suggests some consensus from which to develop a more highly specified and defined model of CRT care than is currently available, although it is currently a model with limited empirical basis.  While not conclusive, there is some empirical basis for recommending that CRTs should provide extended opening hours and include a psychiatrist within the CRT team. Good consensus across qualitative research also suggests CRT managers should prioritise ensuring staff have time to listen to service users’ concerns and not be exclusively task-focused, and should also be able to provide a range of support including help with practical problems. Managers should also seek means to promote continuity and limit the number of different staff a service user sees during an episode of CRT support: one way to achieve this would be to provide each service user with a named worker. The CRT model outlined in government guidance when CRTs were originally mandated in England remains generally supported by the limited available evidence. | P. 12, 14 |
| Limitations | 25 | Due to resource limitations, the web-based search for government and expert guidelines was limited to the UK. Three further limitations of the review should be acknowledged:  Firstly, the wide variation among studies in study design and quality and regarding CRT implementation, outcomes measured, and setting and study populations – together with substantial missing data regarding the characteristics of CRT teams – meant that we could not carry out quantitative synthesis of results from quantitative studies. This limited the direct comparison of the effectiveness of CRTs in different studies. An example of such a synthesis is the meta-regression conducted by Burns and colleagues [101], which usefully identified components of intensive case management services associated with reductions in inpatient bed use.  Secondly, the quality assessment measure used in this review was relatively crude. The retrieval of papers using a mixture of methods meant that the MMAT [29] numerical scale of quality assessment was the most appropriate available means of synthesising quality of evidence. In order to counterbalance subjective elements of scoring, assessment was carried out by two authors and disagreements resolved by a third. However, there are limitations inherent in conducting an assessment of the risk of bias in retrieved papers through the use of a scale that ‘numerically summarise[s] multiple components into a single number’ and therefore reduces evidence of quality to pre-specified categories [26,86]. A further limitation is that the MMAT treats different methodologies as equivalent, for example there is no weighting for RCTs compared to natural experiments. We used the MMAT because, to our knowledge, it is the best available single measure for assessing quality of studies with the range of different methodologies included in our review.  Thirdly, the inclusion of studies with lower quality scores may compromise the strength of conclusions. No formal assessment of selective outcome reporting or publication bias was undertaken; however, the high number of papers in this review with non-significant results suggests that publication bias might not be a problem. We decided to include all studies, regardless of quality, in order to gauge the current evidence base for the implementation of CRTs in adult mental health settings. Conclusions were drawn with reference to the variability of quality scores of the included studies. | P. 12-13 |
| Conclusions | 26 | Overall, the present findings provide considerable evidence about stakeholders’ priorities for CRTs, which are broadly congruent across stakeholder groups. However, our review allows few confident conclusions about the critical components for effectiveness of CRT services, due to the paucity of empirical evidence in the literature. Further research is required to determine elements of best practice that result in effective CRT service provision, including tools to evaluate adherence to a model of good practice. | P. 14 |
| **FUNDING** | | |  |
| Funding | 27 | This paper was written as part of the CORE Study, a research programme funded by the United Kingdom National Institute for Health Research (NIHR) under its Programme Grants for Applied Research programme (Reference Number: RP-PG-0109-10078). The views expressed are those of the authors and not necessarily those of the NHS, the UK NIHR or the UK Department of Health. | P. 16 |

*From:*  Moher D, Liberati A, Tetzlaff J, Altman DG, The PRISMA Group (2009). Preferred Reporting Items for Systematic Reviews and Meta-Analyses: The PRISMA Statement. PLoS Med 6(6): e1000097. doi:10.1371/journal.pmed1000097

For more information, visit: **www.prisma-statement.org**.
