# Supplementary material for: Implementation of the Crisis Resolution Team model in adult mental health settings: a systematic review
Source: BMC Psychiatry. 2015 Apr 8;15:74. doi: 10.1186/s12888-015-0441-x (PMC4405828; doi:10.1186/s12888-015-0441-x)
Supplement: Additional file 2: — Microsoft Word document. Search terms. Table DS1. CRT implementation Review - search terms for electronic database search. Table DS2. CRT implementation review - web resources searched for government and expert organisations’ guidelines. [file 12888_2015_441_MOESM2_ESM.docx]

**Additional file 2**

**Table DS1: CRT implementation Review - search terms for electronic database search**

|  |  | **MEDLINE** | **Embase** | **PsycINFO** | **CINAHL** | **Web of Science** |
| --- | --- | --- | --- | --- | --- | --- |
| **Mental health subject heading** | 1 | Mental Disorders  OR  Mental Health Services | Psychiatry  OR  Mental Health | Mental Disorders | Mental Disorders  OR  Mental health services | Psychiatry |
|  | AND | AND | AND | AND | AND | AND |
| **Terms for CRTs** | 2 | “crisis intervention”  OR  “crisis resolution”  OR  “crisis assessment”  OR  “home treatment”  *(in abstract)*  OR  “Crisis Intervention” *(MeSH term)* | “crisis intervention”  OR  “crisis resolution”  OR  “crisis assessment”  OR  “home treatment”  *(in abstract)* | “crisis intervention”  OR  “crisis resolution”  OR  “crisis assessment”  OR  “home treatment”  *(in abstract)* | “crisis intervention”  OR  “crisis resolution”  OR  “crisis assessment”  OR  “home treatment”  *(in abstract)* | “crisis intervention”  OR  “crisis resolution”  OR  “crisis assessment”  OR  “home treatment”  *(in topic)* |

**Table DS2: CRT implementation review - web resources searched for government and expert organisations’ guidelines**

| **Source [reference]** | **URL and search terms (all searches updated to 30/11/2013)** |
| --- | --- |
| Department of Health Publications archive [A1, A2] | <http://www.dh.gov.uk/en/Publicationsandstatistics/Publications/PublicationsLibrary/index.htm> [link no longer available]  “mental health services” OR “crisis resolution”  [https://www.gov.uk/government/publications?keywords=%22mental%2Bhealth%2Bservices%22&publication_filter_option=all&topics[]=all&departments[]=department-of health&official_document_status=all&world_locations[]=all&from_date=15%2F01%2F2012&to_date=08%2F11%2F2013](https://www.gov.uk/government/publications?keywords=%22mental%2Bhealth%2Bservices%22&publication_filter_option=all&topics%5b%5d=all&departments%5b%5d=department-of%20health&official_document_status=all&world_locations%5b%5d=all&from_date=15%2F01%2F2012&to_date=08%2F11%2F2013)  NB no multi-word searching facility, i.e. the results include the words ‘mental’ or ‘health’ or ‘services’ or ‘crisis’ or ‘resolution’ |
| Centre for Mental Health Publications list [A3] | <http://www.centreformentalhealth.org.uk/publications/publications_list.aspx?SortID=a>  List of all publications searched |
| Kings Fund website Publications page [A4] | <http://www.kingsfund.org.uk/publications/>  “mental health” |
| Rethink website research page [A5] | <http://www.rethink.org/how_we_can_help/research/?shortcut=research>  Reports searched from Rethink studies in 5 research themes listed on website |
| MIND website reports page [A6] | <http://www.mind.org.uk/campaigns_and_issues/report_and_resources/s1>  List of all publications searched |
| Royal College of Psychiatrists website [A7] | <http://rcpsych.ac.uk/publications.aspx>  List searched of all College reports, position statements and occasional papers |
| National Audit Office [A8] | <http://www.nao.org.uk/publications>  “crisis resolution” |
| National Mental Health Development Unit [A9] | <http://www.nmhdu.org.uk/search/> “crisis resolution” [link no longer available] |

A1. Department of Health publications archive (previous version: accessed 05/2012). <http://www.dh.gov.uk/en/Publicationsandstatistics/Publications/PublicationsLibrary/index.htm>

A2. Department of Health publications archive [https://www.gov.uk/government/publications?keywords=%22mental%2Bhealth%2Bservices%22&publication_filter_option=all&topics[]=all&departments[]=department-of health&official_document_status=all&world_locations[]=all&from_date=15%2F01%2F2012&to_date=08%2F11%2F2013](https://www.gov.uk/government/publications?keywords=%22mental%2Bhealth%2Bservices%22&publication_filter_option=all&topics%5b%5d=all&departments%5b%5d=department-of%20health&official_document_status=all&world_locations%5b%5d=all&from_date=15%2F01%2F2012&to_date=08%2F11%2F2013)

A3. Centre for Mental Health publications list. <http://www.centreformentalhealth.org.uk/publications/publications_list.aspx?SortID=a> (current version as of 12/2013).

A4. Kings Fund website publications page. <http://www.kingsfund.org.uk/publications/> (current version as of 12/2013).

A5. Rethink website research page. <http://www.rethink.org/how_we_can_help/research/?shortcut=research> (current version as of 12/2013).

A6. MIND website reports page. <http://www.mind.org.uk/campaigns_and_issues/report_and_resources/s1> (current version as of 12/2013).

A7. Royal College of Psychiatrists website. <http://rcpsych.ac.uk/publications.aspx> (current version as of 12/2013).

A8. National Audit Office. <http://www.nao.org.uk/publications> (current version as of 12/2013).

A9. National Mental Health Development Unit. <http://www.nmhdu.org.uk/search/> (current version as of 12/2013).
