# Supplementary material for: Implementation of the Crisis Resolution Team model in adult mental health settings: a systematic review
Source: BMC Psychiatry. 2015 Apr 8;15:74. doi: 10.1186/s12888-015-0441-x (PMC4405828; doi:10.1186/s12888-015-0441-x)
Supplement: Additional file 3: — Microsoft Word document. Data extraction tables. Table DS3. CRT implementation review - study characteristics: CRTs vs TAU Table DS4. CRT implementation review - study characteristics and findings for CRT surveys. Table DS5. CRT implementation review - study characteristics from CRT stakeholder interviews, questionnaires and surveys. [file 12888_2015_441_MOESM3_ESM.docx]

**Additional file 3**

**Table DS3 CRT implementation review - study characteristics: CRTs vs TAU**

| ***Study Reference*** | **Type of study** | **Setting** | **Number of teams involved** | **Number of participants** | **Study Inclusion/exclusion criteria** | **Duration of study** | **Outcomes assessed** |
| --- | --- | --- | --- | --- | --- | --- | --- |
| *Adesanya (2005)[36]* | Natural experiment (pre- and post- comparisons) | Rural Australia | 2 services:  CRT; versus pre-CRT services | N=122  Pre-CRT n=69  CRT n=53 | Inclusion: All adult admissions over 3 year study period (18 months before and 18 months after CRT) | 3 years, no follow-up periods given | Service use (hospital admission, readmission and bed days) |
| *Barker (2011) [12]* | Natural experiment (pre- and post- comparisons) | Edinburgh, UK | 3 services:  Two CRT teams, in N and S Edinburgh versus pre-CRT services | Total N not stated. Mean yearly admissions pre-CRT  n=1266  CRT n =955 | Inclusion: All acute admissions between 2003-2009 (5 years before and 1 year after CRT) | Follow-up for readmissions was 365 days | Service use (hospital admission, readmissions, MHA admissions and bed days) |
| *Bechdolf (2011)[37]* | Natural experiment (pre- and post- comparisons) | Krefeld, Germany | 2 services:  CRT versus standard care service | N(patients)=753  N(treatments )=1089  CRT n=106 patients, 126 treatment cases  Control n=647 patients, 963 treatment cases | Inclusion: All acute admissions to local CRT and hospital in 2001  Exclusions: SU’s who had received both CRT and hospital care at some point; SU’s with main diagnosis of gerontopsychiatric disorder or addiction | 2 year follow-up | Service use (admission/treatment period) |
| *Dean (1993)[38]* | Natural experiment (prospective study of parallel groups) | Birmingham, UK | 2 services:  CRT service versus standard care services | N=124  CRT n= 69  Comparison n=55 | Inclusion: All adults ‘in crisis’ seen by CRT or standard care between Jan 1990-Feb 1991 | Follow up at 1month and 1 year | Service use (inpatient bed days); service user wellbeing; carer burden |
| *Dibben (2008)[39]* | Natural experiment (pre- and post- comparisons) | West Suffolk | 2 services:  CRT versus pre- CRT services | N=167  Pre-CRT: n=65  CRT: n=102  Satisfaction questionnaires n=84 (service users n=28; carers n=56) | Inclusion: All adults age 65+ in crisis between Sept 2005-6 (6mths before and after CRT)  Exclusion: People detained under Mental Health Act; and out-of-area admissions | 1 year, no follow-up period given | Service use (hospital admissions and bed days);  patient satisfaction |
| *Forbes (2010)[40]* | Natural experiment (pre- and post- comparisons) | Midlothian, Scotland | 2 services:  CRT service versus pre-CRT services | Exact numbers not stated | Inclusion: All adult admissions with a GP in Midlothian, over 2 year study period May 2006- April 2008 (1 year before and 1 year after CRT introduced) | Admissions and bed day data over 2 year period, no follow up | Service use (hospital admissions, bed days);  rates of Mental Health Act interventions |
| *Guo (2001)[41]* | Natural experiment (pre- and post- comparisons) | A Midwestern metropolitan area in the USA | 2 services:  CRT versus pre-CRT services | N=2,200  Pre-CRT n=1,100  CRT n=1,100 | Inclusion: All service users in 11 months of 1995 and 1997 were eligible. The cohorts were matched | Patients tracked for 30 days after initial contact | Service use (admission rates) |
| *Hugo (2002)[42]* | Natural experiment (prospective study of parallel groups) | Suburban Adelaide, Australia | 2 services:  CRT versus pre-CRT services | N=461  CRT n=298  Hospital service n=163 | Inclusion: All clients using emergency crisis services in the 3 month period.  Exclusion: Involuntary hospital admissions | 3 month retrospective cohort | Service use (admission rates) |
| *Jethwa (2007)[43]* | Natural experiment (pre- and post- comparisons) | Leeds, UK | 2 services:  CRT versus pre-CRT treatment as usual | N=4353  Pre-CRT n=3325  CRT n=1028 | Inclusion: All adult hospital admissions over 3 year period (2 years before and 1 year after CRT introduced) | Data collected for 2 years prior and 1 year post-implementation of CRT | Service use (admission rates) |
| *Johnson (2005a)[8]* | Natural experiment (pre- and post- comparisons) | Islington, London, UK | 2 services:  CRT versus pre-CRT treatment as usual | N=200  Pre-CRT n=77  CRT n=123 | Inclusion: All adult 'crisis' presentations between 6 months before and 9 months after the establishment of CRT | Assessments after crisis, at 6 weeks, and at 6 months | Service use (admission rates); patient satisfaction; clinical and social outcomes (symptoms and adverse events) |
| *Johnson (2005b)[9]* | RCT | Islington, London, UK | 2 services:  CRT versus standard care service | N=260  CRT n=135  Control n=125 | Inclusion: Adults experiencing a crisis  Exclusion: Admitted directly to hospital, managed in the community, opted out, unable to consent, no consenting family | Data was collected 8 weeks and 6 months after initial crisis | Service use (admission rates); patient satisfaction; clinical outcomes (symptom severity, adverse events) |
| *Johnson (2008)[44]* | RCT | North Southwalk, London, UK | 2 services:  CRT versus standard care service | N=179  CRT n=91  Control n=88. 97  39 % completed satisfaction Qs | Inclusion: Patients assessed as requiring admission in the catchment area | Initial assessment and 6 months. Satisfaction at 8 weeks | Service use (bed days);  patient satisfaction |
| *Keown (2007)[45]* | Natural experiment (pre- and post- comparisons) | Newcastle and North Tyneside | 2 services:  CRT versus pre-CRT services | N=1517 | Inclusion: All adult admissions in the area in the same 3 month period over 5 years (1999-2004) | Data accessed after a year had passed | Service use (admissions, compulsory detentions, bed days); diagnosis; suicide rates |
| *Kolbjornsrud (2009)[46]* | Natural experiment (pre- and post- comparisons) | Telemark, Norway | 2 services:  CRT versus pre-CRT services | N=1221 | Inclusion: All patients using acute care during the study period | Data collected over 2 years. No follow-up | Service use (admission rates) |
| *Piggott (1993)[47]* | Natural experiment (prospective study of parallel groups) | Connecticut, USA | 2 services:  CRT versus non-CRT services | N=610  CRT n=187  Comparison n=423 | Inclusion: All people in crisis suitable for hospitalisation Jan 1990-Dec 1991  Exclusion: Substance misuse | Data collected for 2 years, no info on follow-up | Service use (admission rates, readmission rates, bed days) |
| *Tyrer (2010)[13]* | Natural experiment (pre- and post- comparisons) | Wales: central Cardiff and the Cardiff suburbs | 4 Services:  CRT versus pre-CRT services | N=1577 | Inclusion: All crises in the area in two 9 month periods: 2004-2005 and 2005-2006 | Service use data collected over 18 months. Satisfaction & functioning assessed after 4 weeks | Service use (admission rates, admission status, bed days); service user satisfaction; service user functioning and quality of life |

**Table DS4: CRT implementation review - study characteristics and findings for CRT surveys**

| **Study reference** | **Quality score from MMAT** | **Type of study** | **Setting** | **Time period** | **Participants** | **Main results** |
| --- | --- | --- | --- | --- | --- | --- |
| Glover (2006) [7] | 3 | Naturalistic observational study | 229 of the 303 local health authorities in England | 1998/9 to 2003/4 | Working age adult patients (under 65 years) of all 229 local health authorities | - 74% of Primary Care Trusts with broadly defined CRTs (any CRT or assertive outreach team) and 83% of narrowly defined CRTs (team on call 24/7) in place by 2001 showed a fall in total admissions, compared with only 60% of those with no team in 2003/4. - For broadly defined CRTs, only the model for women aged 35-64 years showed a significant reduction in admissions at the P<0.001 level. - For restrictively defined CRTs, there was a significant reduction in admissions for older men and women at the P<0.001 level. There was a highly significant reduction in admissions for younger women (p=0.003), but not for younger men (P=0.03). - For restrictively defined CRTs there was a significant reduction in beds for all people (P=0.005), for younger women (P=0.005) and older men (P<0.001). - There was no significant change in bed usage for broadly defined CRTs. - **CRTs which provide a 24/7 service may be more effective than those which don’t in reducing hospital admissions.** |
| Jacobs (2011) [14] | 4 | Secondary analysis of data from Glover et al. (2006) |  |  |  | - The overall CRT effect suggests lower admissions for PCTs with CRT of around 37 admissions, but this difference is not significant. - The authors found no evidence that the CRT policy per se has made any difference to admissions. - **There was no evidence for how CRTs can function most effectively** |
| Hasselberg (2011a) [19] | 4 | Naturalistic study | 8 CRTs (out of the 9 operating at the time of data collection) in Norway | Inclusion period of 3 months in 2005 or beginning of 2006. Period could be prolonged to include 60 patients from each team | 680 patients seen by the 8 CRTs. Participants were 18 years or over and had had face-to-face consultation with the CRT. Number of patients included from each team ranged from 46-147 | - None of the teams were 24/7, none had gate-keeping functions for acute wards, and 40% were waiting for over 24 hours before being seen - Patients with previous contact with mental health services and with emergency referrals had significantly more mental health problems than those without on clinical measures (HoNOS total score, p<.001, GAF symptoms and GAF functioning, p<.001). - Patients who self-referred did not differ significantly on any clinical measures from those who were referred (HoNOS total score p=0.42; GAF symptoms p=0.28; GAF functioning p=0.05). - CRTs with extended opening hours accepted more severely ill patients (HoNOS score p<0.001) than those operating office hours only. - **Limited opening hours may reduce CRTs’ ability to work with severely ill patients.** - **Accepting self-referrals may not dilute CRTs’ focus on working with severely ill patients.** |
| Hasselberg (2011b) [48] | 4 | Naturalistic study using the same cohort as Hasselberg et al. 2011a |  |  |  | - The overall symptom improvement after CRT care was positive, with small to medium effect size (*d* = 0.15-0.45 across the GAF and HoNOS total scores). - Length of treatment (p < 0.001); being male (p = 0.002); being single (p = 0.013); and a team focus on out-of-office contact (p = 0.016) were predictors of favourable outcomes of crises in the adjusted model. - **Treatment at home may lead to better outcomes after crisis.** - **Longer treatment periods may lead to better outcomes.** |

**Table DS5: CRT implementation review - study characteristics from CRT stakeholder interviews, questionnaires and surveys**

*SU=service user; C=carer; CRT=Crisis Resolution Team; GP=General Practitioner; P=participant*

| **Study reference** | **Quality score from MMAT** | **Setting** | **No. of CRTs** | **Details of CRT(s)** | **No. of P’s** | **P’s inclusion criteria/**  **characteristics** | **Type of study** |
| --- | --- | --- | --- | --- | --- | --- | --- |
| ‘Amaze’, Shaw (2010) [49] | 3 | Nottinghamshire and Lincolnshire, UK | 9 | N/A | 36 | SU’s of CRTs during time-frame | Semi-structured face to face interviews |
| Ampélas (2005) [50] | 4 | France | 1 | ‘ERIC’; more emphasis on treatment over the phone; 24/7 telephone service | 81 + 73 | 81 carers and 73 SU’s | Questionnaires with answers reported back by telephone |
| Armitage (2006) [51] | n/a | Leicester City, UK | 1 | ‘Leicester City CRT’, 24/7; Catchment area ~Leicester City | 470 | SU’s | Face to face survey |
| Borg (2010) [52] | 2 | Norway | 1 | Multi-disciplinary team; uses open dialogue and lifeworld approach | 12 | CRT members: 1 psychologist, 2 social workers, 9 nurses | Focus groups |
| Freeman (2011) [53] | 3 | South Wales, UK | 1 | Catchment area: 250,000 | 5 | CRT staff members (mean time worked in CRT: 2 years 5 months) | Semi-structured face to face interview |
| Fulford (2001) [54] | 2 | Nothern and Central Eastern areas of Melbourne, Australia | 1 | ‘CAT’ team, longer max treatment (8 weeks) | 77 | Carers | Quantitative data questionnaire (+ minor qualitative elements) |
| Hannigan (2010) [55] | 4 | Northtown, Wales,UK | 1 | ‘Lakeside’ team | 34 | CRT staff members | Case study including semi-structured interviews and observation of team meetings |
| Hopkins (2007) [16] | 2 | Newcastle and North Tyneside, UK | 1 | CRT ‘CATS’; catchment area: 450,000 | 21 | SU’s | Semi-structured face to face interviews |
| Karlsson (2008) [56] | 1 | Norway | 1 | CRT uses ‘open dialogue’ | 7 | SU’s | Case study involving face to face interviews |
| Khalifeh (2009) [57] | 3 | Camden and Islington boroughs of London, UK | 4 | Area with highest level of morbidity in UK; Catchment area: 373,817 | 18 + 5 | 18 SU’s (all mothers) + 5 children of some SU’s | Semi-structured face to face interviews |
| Lyons (2009) [58] | 2 | Lancashire, UK | 1 | Data collected from CRT in 2006 | 471 | Stakeholders in CRT (carers, current and previous SU’s, staff, managers, service commissioners) | Postal questionnaires (220 P’s) and group discussions (251 P’s; 24 group meetings) |
| McCauley (2005) [59] | 2 | Cavan, North East Ireland | 1 | Rural area (population ~ 56,000); multidisciplinary team, with psychiatrists | 30 | GPs who had referred SU’s to CRT | Face to face questionnaire |
| Middleton (2011) [60] | 3 | East Midlands, UK | 9 | Catchment area (of all 9): 1.2 million | 36 | SU’s currently in treatment | Face to face interview |
| MIND (2011) [17] | n/a | England and Wales, UK | N/A | N/A | 350 | SU’s, carers, staff, poll on website; (teams of experts generated proposals based on data) | Internet survey, meetings and visits |
| Morgan (2008) [61] | 0 | England, UK | 25 | N/A | 50 | 25 CRT managers and 25 ward managers | Structured interviews |
| Morton (2009) [62] | 4 | North West of England, UK | 1 | Mostly urban boroughs | 27 | CRT staff members, on current case studies | Structured face to face interviews |
| NAO (2007) [63] | n/a | England, UK | 54 | Wards and CRTs nationwide  (Data collected 2006/7) | 25 + 29 | 25 ward managers and representatives of CRT’s interviewed on last 20 cases; 29 CRT’s provided data themselves | Audit (focus groups, surveys, interviews) |
| Nelson (2009) [64] | 3 | London (UK) boroughs: Camden, Islington, Southwark Lambeth, Waltham Forest, Kensington and Chelsea | 11 | Mean time of existence 3.5 years | 132 | CRT staff members | Postal questionnaires |
| Onyett (2008) [4] | 3 | England, UK | 177 | CRT’s nationwide  (Data collected 2005/6) | 177 | CRT’s staff members | Survey (online questionnaires and subsequent interviews) |
| Reynolds (1990) [65] | 3 | New South Wales, Australia | 1 | Data collected from CRTs 1986/7 | 69 + 50 | SU’s, only acute patients (69); and carers (50) | Qualitative and quantitative data questionnaires and subsequent interviews (55 P’s only) |
| Taylor 2012 [66] | 2 | Not stated (London?) | 1 | Access to Mental Health Services Team  (AT) introduced in April 2001 to provide a 24-hour crisis resolution and home treatment service | 49 | SU’s referred to the CRT in 4 month time period | Qualitative semi-structured interviews (face-to-face or telephone); postal questionnaire |
| Tobitt (2011) [67] | 4 | Sussex, UK | 4 | Urban and rural areas | 39 | CRT staff members | Semi-structured face to face interview |
| Wasylenki (1997) [68] | 1 | Toronto, Canada | 1 | Local mental health services experimented implementation of CRT | 27 (+ 44) | 27 SU’s; crisis sufferers underwent CRT, then reported on experiences; 44 experts interviewed | Experiment and self-report |
| Weich (2012) [69] | 4 | Birmingham, UK | 3 | Home Treatment teams covered by a single Primary Care Trust | 53 | SUs accepted by HTT with clinical diagnosis of psychotic disorder | Semi-structured face to face interview |

**Table DS6: CRT implementation review - government and expert guidelines included in review**

| **Title** | **Source** | **Publication Date** | **Description** |
| --- | --- | --- | --- |
| The NHS Performance Framework: Implementation Guide [70] | Department of Health, NHS Finance, Performance & Operations Directorate | 2011 | Supports the application of the NHS Performance Framework in England, and informs mental health trusts of the criteria against which their performance will be assessed |
| Mental Health and the Productivity Challenge [71] | The King’s Fund and The Sainsbury Centre for Mental Health (Eds. Naylor and Bell) | 2010 | Seeks to demonstrate there is scope to improve productivity in mental health care to help the NHS meet the target of 4% spending improvement each year for the next four years. Points to reducing unnecessary bed use in acute care by strengthening CRTs as a promising money saving target |
| Getting the Medicines Right 2: Medicines Management in Mental Health and Crisis Resolution and Home Treatment Teams [74] | National Mental Health Development Unit  (Eds. Davies P, Taylor J) | 2010 | Focuses on best practice for medicines management following an extensive review of clinical practices. Commissioned by the NMHDU Acute Care Board. Findings in this report were supported by a National Survey conducted across all mental health trusts in England |
| Mental Health and Social Inclusion [72] | Royal College of Psychiatrists Social Inclusion Scoping Group | 2009 | Position statement produced by the Social Inclusion Scoping Group of the Royal College of Psychiatrists. The Scoping groups was set up to examine the nature and extent of social exclusion among people with mental health problems and those with intellectual disabilities and the implications for the future practice and training of psychiatrists |
| Helping People Through Mental Health Crisis: The Role of Crisis Resolution and Home Treatment Services [73] | National Audit Office  (Report by the Controller and Auditor General) | 2007 | The NAO reviewed data collected by CRTs/Trusts on admissions, costs, service user and carer satisfaction, and also conducted their own survey plus focus groups for CRT service users and their carers |
| New Ways of Working for Everyone: A Best Practice Guide [75] | Department of Health | 2007 | Best practice implementation guide to set out how health and social care organisations could take a strategic approach to the implementation of ‘New Ways of Working’ |
| Guidance Statement on Fidelity and Best Practice for Crisis Services [76] | Department of Health  (Eds. Crompton N, Daniel D) | 2007 | Identifies aspects of how crisis teams deliver care, which need attention so that best practice is followed across England |
| Crisis Resolution and Home Treatment: A Practical Guide [77] | Sainsbury Centre for Mental Health  (Ed. McGlynn P. Authors: Bridgett C, Flowers M, Ford K, Hoult J, Lakhani N, McGlynn P, Woodbridge K) | 2006 | Draws on the authors’ experiences of delivering training and practice development to CRT teams and attempts to place the implementation of these teams within the wider framework of development and modernisation of services in the England |
| Beyond the Water Towers: The Unfinished Revolution in Mental Health Services 1985-2005 [78] | Sainsbury Centre for Mental Health  (Eds. Bell A & Lindley P) | 2005 | Provides an overview of the developments in mental health care during the 20 years the Sainsbury Centre has been in existence. Makes recommendations for improving mental health services going forward, although the suggestions are general, not specific to CRTs |
| Transforming Mental Health Care: Assertive Outreach and Crisis Resolution in Practice [79] | Sainsbury Centre for Mental Health  (Eds. Chisholm A & Ford R) | 2004 | Guide to the development of assertive outreach and crisis resolution services based on the experience of new and existing teams across England |
| Carers Under Pressure [80] | Rethink | 2003 | Publication exploring the impact caring has on the individual carer and what kinds of relationships with mental health services most benefit carers |
| The Mental Health Policy Implementation Guide [2] | Department of Health | 2001 | Supports the delivery of adult mental health policy in England |
| Setting up and Running Crisis Resolution Services [81] | Sainsbury Centre for Mental Health | 2001 | Provides guidance to local trusts needing to set up new CRTs in response to the NHS Plan released in July 2000, which called for the creation of 335 CRTs over 3 years in the UK |
| Mental Health National Service Framework: Workforce Planning, Education, and Training [82] | Workforce Action Team for NHS | 2001 | A publication by the Workforce Action Team, a workgroup set up to support the implementation of one of the five underpinning programmes of the National Service Framework and NHS plan |
| The Triangle of Care. Carers Included: A Guide to Best Practice in Acute Mental Health Care [83] | National Mental Health Development Unit  (Eds. Worthington A & Rooney P) | 2010 | Identifies 6 key elements required to achieve better collaboration and partnership with carers in the service user and carer’s journey through an acute episode and how to achieve these elements |
| Service User Experience in Adult Mental Health [84] | National Collaborating Centre for Mental Health | 2012 | NICE-commissioned guidance for secondary mental health services on achieving a high-quality service user experience, including crisis services |
| Guidance for Commissioners of Acute Care: Inpatient and Crisis Home Treatment [6] | Joint Commissioning Panel for Mental Health (JCPMH) | 2013 | Commissioning guidance to raise quality in services including crisis teams |
| Completing the Revolution: Transforming Mental Health and Tackling Poverty [85] | The Centre for Social Justice | 2011 | Review into the prevalence of mental ill-health among people living in challenging circumstances, and how services including crisis teams can work to minimise this |
| Psychosis and Schizophrenia in Adults: Treatment and Management [5] | NICE | 2014 | Updated NICE guidelines incorporating advice on crisis treatment and referral for adults with psychosis and schizophrenia |
| The Abandoned Illness [86] | The Schizophrenia Commission (established by Rethink Mental Illness) | 2014 | Inquiry into and guidance on mental health service provision for people with schizophrenia |
