# Supplementary material for: Implementation of the Crisis Resolution Team model in adult mental health settings: a systematic review
Source: BMC Psychiatry. 2015 Apr 8;15:74. doi: 10.1186/s12888-015-0441-x (PMC4405828; doi:10.1186/s12888-015-0441-x)
Supplement: Additional file 4: — Microsoft Word document. MMAT scoring for studies included in this review. Table DS7. CRT implementation review - MMAT scores for studies comparing two CRTs. Table DS8. CRT implementation review - MMAT scores for studies comparing a CRT with TAU. Table DS9. CRT implementation review - MMAT scores for CRT surveys. Table DS10. CRT implementation review - MMAT scores for stakeholder interviews, surveys and questionnaires. [file 12888_2015_441_MOESM4_ESM.docx]

**Additional File 4**

**Table DS7: CRT implementation review - MMAT scores for studies comparing two CRTs**

| **Types of mixed methods study components or primary studies** | **Methodological quality criteria (see tutorial for definitions and examples)** | Allen (2009) [31] | Doyle (1994) [32] | Happell (2009) [33] | Harrison (2011) [34] | Reding, (1995) [35] |
| --- | --- | --- | --- | --- | --- | --- |
| **Screening questions (for all types)** | Are there clear qualitative and quantitative research questions (or objectives*), or a clear mixed methods question (or objective*)? | N | Y | Y | Y | Y |
|  | Do the collected data address the research question (objective)? Eg, consider whether the follow-up period is long enough for the outcome to occur (for longitudinal studies or study components). | N | Y | Y | Y | Y |
| **3. Quantitative non-randomized** | 3.1. Are participants (organizations) recruited in a way that minimizes selection bias? | n/a | N | Y | Y | Y |
|  | 3.2. Are measurements appropriate (clear origin, or validity known, or standard instrument; and absence of contamination between groups when appropriate) regarding the exposure/intervention and outcomes? | n/a | Y | Y | Y | Y |
|  | 3.3. In the groups being compared (exposed vs. non-exposed; with intervention vs. without; cases vs. controls), are the participants comparable, or do researchers take into account (control for) the difference between these groups? | n/a | Y | Y | N | Y |
|  | 3.4. Are there complete outcome data (80% or above), and, when applicable, an acceptable response rate (60% or above), or an acceptable follow-up rate for cohort studies (depending on the duration of follow-up)? | n/a | Y | N | Y | Y |
|  | TOTALS | n/a | 3 | 3 | 3 | 4 |

**Table DS8: CRT implementation review - MMAT scores for studies comparing a CRT with TAU**

| **Types of mixed methods study components or primary studies** | **Methodological quality criteria (see tutorial for definitions and examples)** | Adesanya (2005) [36] | Barker (2011) [12] | Bechdolf (2011) (German) [37] | Dean(1993) [38] | Dibben (2008) [39] | Forbes (2010 )[40] | Guo (2001) [41] | Hugo (2002) [42] | Jethwa (2007) [43] | Johnson (2005a) [8] | Johnson (2005b) [9] | Johnson (2008) [44] | Keown (2007) [45] | Kolbjornsrud (2009) (Norwegian) [46] | Pigott (1993) [47] | Tyrer (2010) [13] |
| --- | --- | --- | --- | --- | --- | --- | --- | --- | --- | --- | --- | --- | --- | --- | --- | --- | --- |
| **Screening questions (for all types)** | Are there clear qualitative and quantitative research questions (or objectives*), or a clear mixed methods question (or objective*)? | Y | Y | Y | Y | Y | Y | Y | Y | Y | Y | Y | (Y) | Y | Y | Y | Y |
|  | Do the collected data address the research question (objective)? Eg, consider whether the follow-up period is long enough for the outcome to occur (for longitudinal studies or study components). | Y | Y | Y | Y | Y | Y | Y | Y | Y | Y | Y | (Y) | Y | Y | Y | Y |
| **1. Qualitative** | 1.1. Are the sources of qualitative data (archives, documents, informants, observations) relevant to address the research question (objective)? |  | Y |  |  |  |  |  |  |  |  |  |  |  |  |  |  |
|  | 1.2. Is the process for analyzing qualitative data relevant to address the research question? |  | N |  |  |  |  |  |  |  |  |  |  |  |  |  |  |
|  | 1.3. Is appropriate consideration given to how findings relate to the context, eg the setting, in which the data were collected? |  | N |  |  |  |  |  |  |  |  |  |  |  |  |  |  |
|  | 1.4. Is appropriate consideration given to how the findings relate to researchers’ influence, eg, through their interactions with participants? |  | Y |  |  |  |  |  |  |  |  |  |  |  |  |  |  |
| **2. Quantitative randomized controlled (trials)** | 2.1. Is there a clear description of the randomization (or an appropriate sequence generation)? |  |  |  |  |  |  |  |  |  |  | Y | (N) |  |  |  |  |
|  | 2.2. Is there a clear description of the allocation concealment (or blinding when applicable)? |  |  |  |  |  |  |  |  |  |  | N | (N) |  |  |  |  |
|  | 2.3. Are there complete outcome data (80% or above)? |  |  |  |  |  |  |  |  |  |  | Y | (N) |  |  |  |  |
|  | 2.4. Is there low withdrawal/drop-out (below 20%)? |  |  |  |  |  |  |  |  |  |  | Y | (N) |  |  |  |  |
| **3. Quantitative non-randomized** | 3.1. Are participants (organizations) recruited in a way that minimizes selection bias? | Y | Y | Y | Y | Y | Y | Y | Y | Y | Y |  |  | Y | Y | Y | Y |
|  | 3.2. Are measurements appropriate (clear origin, or validity known, or standard instrument; and absence of contamination between groups when appropriate) regarding the exposure/intervention and outcomes? | Y | Y | Y | Y | Y | Y | Y | Y | Y | Y |  |  | Y | Y | Y | Y |
|  | 3.3. In the groups being compared (exposed vs. non-exposed; with intervention vs. without; cases vs. controls), are the participants comparable, or do researchers take into account (control for) the difference between these groups? | Y | N | Y | Y | Y | N | Y | Y | N | Y |  |  | Y | Y | Y | N |
|  | 3.4. Are there complete outcome data (80% or above), and, when applicable, an acceptable response rate (60% or above), or an acceptable follow-up rate for cohort studies (depending on the duration of follow-up)? | Y | Y (excluding the qualitative part, which is N) | Y | N | N | Y | Y | Y | Y | N |  |  | Y | Y | Y | N |
| 5. Mixed methods | 5.1. Is the mixed methods research design relevant to address the qualitative and quantitative research questions (or objectives), or the qualitative and quantitative aspects of the mixed methods question (or objective)? |  | Y |  |  |  |  |  |  |  |  |  |  |  |  |  |  |
|  | 5.2. Is the integration of qualitative and quantitative data (or results*) relevant to address the research question (objective)? |  | Y |  |  |  |  |  |  |  |  |  |  |  |  |  |  |
|  | 5.3. Is appropriate consideration given to the limitations associated with this integration, eg, the divergence of qualitative and quantitative data (or results*) in a triangulation design? |  | N |  |  |  |  |  |  |  |  |  |  |  |  |  |  |
|  | TOTALS | 4 | 2 | 4 | 3 | 3 | 3 | 4 | 4 | 3 | 3 | 3 | 0 | 4 | 4 | 4 | 2 |

**Table DS9: CRT implementation review - MMAT scores for CRT surveys**

| **Types of mixed methods study components or primary studies** | **Methodological quality criteria (see tutorial for definitions and examples)** | Glover (2006) [7] | Hasselberg (2011a) (Norway) [19] | Jacobs (2011) [14] | Hasselberg (2011b) (Norway) [48] |
| --- | --- | --- | --- | --- | --- |
| **Screening questions (for all types)** | Are there clear qualitative and quantitative research questions (or objectives*), or a clear mixed methods question (or objective*)? | Y | Y | Y | Y |
|  | Do the collected data address the research question (objective)? Eg, consider whether the follow-up period is long enough for the outcome to occur (for longitudinal studies or study components). | Y | Y | Y | Y |
| **3. Quantitative non-randomized** | 3.1. Are participants (organizations) recruited in a way that minimizes selection bias? | Y |  | Y |  |
|  | 3.2. Are measurements appropriate (clear origin, or validity known, or standard instrument; and absence of contamination between groups when appropriate) regarding the exposure/intervention and outcomes? | Y |  | Y |  |
|  | 3.3. In the groups being compared (exposed vs. non-exposed; with intervention vs. without; cases vs. controls), are the participants comparable, or do researchers take into account (control for) the difference between these groups? | N |  | Y |  |
|  | 3.4. Are there complete outcome data (80% or above), and, when applicable, an acceptable response rate (60% or above), or an acceptable follow-up rate for cohort studies (depending on the duration of follow-up)? | Y |  | Y |  |
| **4. Quantitative descriptive** | 4.1. Is the sampling strategy relevant to address the quantitative research question (quantitative aspect of the mixed methods question)? |  | Y |  | Y |
|  | 4.2. Is the sample representative of the population understudy? |  | Y |  | Y |
|  | 4.3. Are measurements appropriate (clear origin, or validity known, or standard instrument)? |  | Y |  | Y |
|  | 4.4. Is there an acceptable response rate (60% or above)? |  | Y |  | Y |
|  | TOTALS | 3 | 4 | 4 | 4 |

**Table DS10: CRT implementation review - MMAT scores for stakeholder interviews, surveys and questionnaires**

| **Types of mixed methods study components or primary studies** | **Methodological quality criteria (see tutorial for definitions and examples)** | Amaze (Shaw) (2010) [49] | Ampelas (2005) [50] | Armitage (2006) [51] | Borg (2010) (Norway) [52] | Freeman (2011) [52] | Fulford (2001) (Australia) [54] | Hannigan (2010) [55] | Hopkins (2007) [16] | Karlsson (2008) (Norway) [56] | Khalifeh (2009) [57] | Lyons (2009) [58] | McCauley (2005) [59] | Middleton (2011)^[[1]](#endnote-1)^ [60] | MIND (2011) [17] | Morgan (2008) [61] | Morton (2009) [62] | NAO (2007) (Part 3, p. 20) [63] | Nelson (2009) [64] | Onyett (2008) [4] | Reynolds (1990) [65] | Taylor (2012) [66] | Tobitt (2011) [67] | Wasylenki (1997) [68] | Weich (2012) [69] |
| --- | --- | --- | --- | --- | --- | --- | --- | --- | --- | --- | --- | --- | --- | --- | --- | --- | --- | --- | --- | --- | --- | --- | --- | --- | --- |
| **Screening questions (for all types)** | Are there clear qualitative and quantitative research questions (or objectives*), or a clear mixed methods question (or objective*)? | Y | Y | N | Y | Y | Y | Y | Y | Y | Y | Y | Y | Y | N | Y | Y | N | Y | Y | Y | Y | Y | Y | Y |
|  | Do the collected data address the research question (objective)? Eg, consider whether the follow-up period is long enough for the outcome to occur (for longitudinal studies or study components). | Y | Y | N | Y | Y | Y | Y | Y | Y | Y | Y | Y | Y |  | Y | Y | n/a | Y | Y | Y | Y | Y | Y | Y |
| **1. Qualitative** | 1.1. Are the sources of qualitative data (archives, documents, informants, observations) relevant to address the research question (objective)? | Y | Y |  | Y | Y |  | Y | Y | N | Y | Y | Y | Y |  | Y |  | n/a |  |  | Y | Y | Y | Y | Y |
|  | 1.2. Is the process for analyzing qualitative data relevant to address the research question? | Y | Y |  | Y | Y |  | Y | Y | Y | Y | Y | Y | Y |  | N |  | N |  |  | N | Y | Y | N | Y |
|  | 1.3. Is appropriate consideration given to how findings relate to the context, eg the setting, in which the data were collected? | N | Y |  | N | Y |  | Y | N | N | Y | N | N | N |  | N |  | N |  |  | Y | N | Y | N | Y |
|  | 1.4. Is appropriate consideration given to how the findings relate to researchers’ influence, eg, through their interactions with participants? | Y | Y |  | N | N |  | Y | N | N | N | N | N | Y |  | N |  | N |  |  | Y | N | Y | N | Y |
| **3. Quantitative non-randomized** | 3.1. Are participants (organizations) recruited in a way that minimizes selection bias? |  |  |  |  |  |  |  |  |  |  |  |  |  |  |  |  |  | N |  |  |  |  |  |  |
|  | 3.2. Are measurements appropriate (clear origin, or validity known, or standard instrument; and absence of contamination between groups when appropriate) regarding the exposure/intervention and outcomes? |  |  |  |  |  |  |  |  |  |  |  |  |  |  |  |  |  | Y |  |  |  |  |  |  |
|  | 3.3. In the groups being compared (exposed vs. non-exposed; with intervention vs. without; cases vs. controls), are the participants comparable, or do researchers take into account (control for) the difference between these groups? |  |  |  |  |  |  |  |  |  |  |  |  |  |  |  |  |  | Y |  |  |  |  |  |  |
|  | 3.4. Are there complete outcome data (80% or above), and, when applicable, an acceptable response rate (60% or above), or an acceptable follow-up rate for cohort studies (depending on the duration of follow-up)? |  |  |  |  |  |  |  |  |  |  |  |  |  |  |  |  |  | Y |  |  |  |  |  |  |
| **4. Quantitative descriptive** | 4.1. Is the sampling strategy relevant to address the quantitative research question (quantitative aspect of the mixed methods question)? |  | Y |  |  |  | Y |  |  |  |  |  | Y |  |  | N | Y |  |  | Y | Y |  |  | Y |  |
|  | 4.2. Is the sample representative of the population understudy? |  | Y |  |  |  | N |  |  |  |  |  | N |  |  | N | Y |  |  | Y | Y |  |  | Y |  |
|  | 4.3. Are measurements appropriate (clear origin, or validity known, or standard instrument)? |  | Y |  |  |  | Y |  |  |  |  |  | N |  |  | Y | Y |  |  | N | N |  |  | Y |  |
|  | 4.4. Is there an acceptable response rate (60% or above)? |  | Y |  |  |  | N |  |  |  |  |  | Y |  |  | Y | Y |  |  | Y | Y |  |  | N |  |
| 5. Mixed methods | 5.1. Is the mixed methods research design relevant to address the qualitative and quantitative research questions (or objectives), or the qualitative and quantitative aspects of the mixed methods question (or objective)? |  | Y |  |  |  |  |  |  |  |  |  | Y |  |  | Y |  |  |  |  | Y |  |  | Y |  |
|  | 5.2. Is the integration of qualitative and quantitative data (or results*) relevant to address the research question (objective)? |  | Y |  |  |  |  |  |  |  |  |  | Y |  |  | Y |  |  |  |  | Y |  |  | Y |  |
|  | 5.3. Is appropriate consideration given to the limitations associated with this integration, eg, the divergence of qualitative and quantitative data (or results*) in a triangulation design? |  | Y |  |  |  |  |  |  |  |  |  | N |  |  | N |  |  |  |  | N |  |  | N |  |
|  | TOTALS | 3 | 4 | n/a | 2 | 3 | 2 | 4 | 2 | 1 | 3 | 2 | 2 | 3 | n/a | 0 | 4 | n/a | 3 | 3 | 3 | 2 | 4 | 1 | 4 |

1. [↑](#endnote-ref-1)
