# Supplementary material for: Implementation of the Crisis Resolution Team model in adult mental health settings: a systematic review
Source: BMC Psychiatry. 2015 Apr 8;15:74. doi: 10.1186/s12888-015-0441-x (PMC4405828; doi:10.1186/s12888-015-0441-x)
Supplement: Additional file 5: — Microsoft Word document. Tables of results and CRT characteristics. Table DS11. CRT implementation review - team characteristics and study outcomes for CRTs compared to TAU. Table DS12. CRT implementation review - CRTs versus other services: Further details of service characteristics. Table DS13. CRT implementation review - Stakeholders’ views on CRTs: themes and recommendations from included. Table DS14: CRT implementation review - recommendations from included government and expert guidelines. [file 12888_2015_441_MOESM5_ESM.docx]

**Additional file 5**

**Table DS11: CRT implementation review - team characteristics and study outcomes for CRTs compared to TAU**

| **Study Ref.** | **Other reported CRT characteristics** | **Characteristics of comparison service** | **Results – service use** |
| --- | --- | --- | --- |
| Adesanya (2005) [36] | The CRT maintained a three-bedroom respite accommodation in the community | Acute inpatient service + non-acute community services | **Service use:** Although statistically non-significant (p=0.2), the results suggest there were proportionately fewer index admissions after the CRT was set up (n=53) than before (n=69). The CRT did not have a significant (p=0.25) impact on the duration of hospitalisation. Prior to CRT mean duration of hospital stay was 35.7 days, after the establishment of CRT it was 33.5 days |
| Barker (2011) [12] | Self-referrals were not accepted but referrals from all other agencies were | Acute inpatient service + non-acute community services | **Service use:** There was a significant decrease in admissions after the CRT was established. Prior to the CRT the mean number of yearly in-patient admissions was 1266. In the year following the introduction of the CRT there were 955 admissions, a 24% decrease (unpaired t-test, p<0.0001).  Length of stay in hospital reduced by 9.21 days (28.58%) from a mean length of stay of 32.23 days between Nov 2004-Nov 2008 to 23.02 days in the year from Nov 2008-Nov 2009. An unpaired t-test showed this to be a significant difference (p<0.0001).  The mean readmission rate in the 5 years before CRT introduction was 348 compared with 282 following introduction. This difference was found to be non-significant when using the un-paired t-test (4% reduction, p=0.152).  **Satisfaction:** In the year following CRT there was a 17% reduction in patients detained under the MHA compared with the 12 months prior to its introduction but this was not found to be statistically significant (p=0.335, x2= 0.93, d.f.=1) using x2 with Yates' correction |
| Bechdolf (2011) [37] |  | Acute inpatient service | **Service use**: CRT had shorter treatment period than hospital; 17,9% of service users broke off CRT treatment |
| Dean (1993) [38] | The service is based in a resource centre in the centre of the locality and is the base for all social services and health services staff. There is a community worker dedicated to helping with employment and many of the staff speak Asian languages. Leisure activities and a drop-in cafe are provided from the centre. The centre gives advice on housing and finance and there are facilities for bathing and laundry. Consumer satisfaction is continually assessed | A hospital service with access to a day hospital based on site + a community rehab service and two community psychiatric nurses | **Service use:** Thirty five % of CRT patients spent some time in hospital during their initial episode. However, they spent significantly fewer days in hospital than those in the control area during this time. The CRT average was 8.3 days (SD 19.88) compared to 58.7 days (SD 95.1)in the control area (z-7.08, p<0.001).  The CRT patients had an average of 20.6 (SD 53.7) days inpatient treatment in the year following crisis, compared with 67.9 (SD 98.7) days in the comparison site (mean ranks z-6.7, p<0.001).  **Other outcomes:** Relatives of the CRT patients were less distressed by their burden at the initial assessment than relatives of Small Heath patients (mean score 0.24 [SD0.29] v 0.36 [SD 0.30], p<0.01).  Relative's distress due to the social performance of the person in treatment was less in the CRT group at one month (mean=0.16, SD 0.31) than in the comparison group (mean= 0.26, SD 0.38). This difference was significant (p<0.01).  At one month more relatives were very satisfied with the care their family member had received in the CRT group than in the comparison service. Twenty two were very satisfied in the CRT group Vs 9 in the comparison service (p<0.05), but by one year this was no longer evident.  Social and psychiatric state of service users a year after starting treatment were similar in the two groups, although both groups improved; there was no significant difference in the total present state examination score (10.3 for Sparkbrook and 9.61 for Small Heath). There was no difference on the general health Q score of the relatives between the two groups at initial assessment, at one month, or one year |
| Dibben (2008) [39] | There was an existing CRT in the area for 18-64yr olds. This study looks at the extension of that service to people aged 65+. This coincided with other service changes such as the closure of a dementia care ward and two day hospitals, and the introduction of an old age intermediate care team | Acute inpatient service + five adult and five older-age community mental health teams in the area | **Service use:** The CRT significantly reduced admissions. In the 6 months pre CRT there were 65 crisis events which resulted in 65 hospital admissions. In the 6 months post there were 102 crisis events of which only 70 required admissions (p<0.001). Overall admissions were reduced by 31%.  There was no significant difference in the length of hospital stay as compared before and after the introduction of the service.  **Other outcomes:** Number of service users detained under the MHA remained the same pre and post CRT (2 x n=9) |
| Forbes (2011) [40] | The CRT service was open between 08.00h and 24.00h, 7 days a week. The team included an occupational therapist and had ready access to social work, psychology and physiotherapy. Consultant care was allocated on the basis of GP service used | A 19-bed inpatient ward was used as part of community mental health. This unit was closed and replaced by allocation of 8 beds 10 miles away and the introduction of the CRT in 2007 | **Service use:** Rates of admission to hospital were unchanged (exact numbers and analysis of data not given). Length of stay increased by a very small amount after the CRT was introduced. Mean length of stay was 18 nights in the 2006-7 period and 19 nights in the 2007-8 period (no analysis).  **Other:** There was an increase in episodes of MHA detentions, it is not mentioned if this was significant or not |
| Guo (2001) [41] | The psychiatrists and nurses provided medical services and conducted diagnostic assessments | Acute inpatient service + hospital-based psychiatric emergency room. No details of non-acute community services | **Service use:** The study found that 32.6 % of the hospital-based crisis cohort were hospitalised within 30 days of initial-crisis contact, as compared with 24.2% of the community-based cohort (x2=263.23, df =33, p<.001). "When all other independent variables were controlled for, a consumer from the hospital-based intervention group was 51% more likely to be hospitalized than a consumer in the community-based mobile crisis intervention group (p<.001)" |
| Hugo (2002) [42] | The team provided assessment, crisis interventions, community based short-term treatment, home visiting and telephone triage services | Acute inpatient service + 24/7 hospital-based psychiatric emergency room (staffed by nurses + doctors). A range of non-acute community services available | **Service use:** Significantly more patients using the hospital based service were admitted to hospital than those using the CRT. Forty three % (n=64) of hospital based contacts and 13% (n=38) of mobile service contacts were admitted to hospital (x2 (1) = 42.98, p<0.001) |
| Jethwa (2007) [43] | Intensive home-based treatment was offered for up to 25 service users at a time. Care plans were agreed specifying the frequency of home visits. The team had access to five pre-existing locally based acute community day service teams and a community respite bed | Acute inpatient service + hospital-based psychiatric liaison service. Non-acute community mental health teams | **Service use:** Generalised linear analysis found a 37.5% reduction in monthly admissions after the introduction of the CRT (p<0.0001). The median number of monthly admissions prior to the team was 140.5, compared with 86.5 after implementation |
| Johnson (2005) [8] | The service conformed to the model described in National Policy Guidelines (Department of Health, 2001). Patients could be visited several times a day | There were acute inpatient wards, two 24 hour staffed crisis houses and a multidisciplinary liaison team available from 8am-10pm in the casualty department + well established community mental health teams available 9am-5pm weekdays | **Service use:** Following introduction of the CRT, the admission rate in the six weeks after a crisis fell significantly from 71% (n-55) to 49% (n=60) (OR 0.38, 95% CI 0.21-0.70, p<0.002). By six months 75% of service users from the pre CRT group had been admitted as compared with 60% of the group who presented after the CRT had been established (p=0.029).  There was a 6.2-day difference in mean bed use between the groups, Wilcoxon’s rank sum test indicated a significant difference between the groups (P=0.0034).  **Satisfaction:** A highly significant difference was found in mean and median patient satisfaction scores. A difference of 5.6 points (95%, CI 2.0-8.3) on mean client satisfaction questionnaire favoured the CRT (p<0.0005).  **Other outcomes:** No clear difference emerged in involuntary hospitalisations, symptoms, social functioning or quality of life |
| Johnson (2005b) [9] | The CRT augmented existing services and aimed to assess all patients and manage them from home if possible | Inpatient unit, crisis houses, and community mental health team | **Service use:** Patients in the experimental group (CRT) were significantly less likely than those in the control to be admitted during the 8 weeks after the crisis (odds ratio 0.19, 95% confidence interval 0.11 to 0.32), though compulsory admission was not significantly reduced. This effect persisted after 6 months.  **Satisfaction:** A difference of 1.6 points in the mean score on the client  satisfaction questionnaire (CSQ-8) was significant after adjustment for baseline  characteristics (P = 0.002) |
| Johnson (2008) [44] |  | Acute inpatient service + 9am-5pm community mental health teams | **Service use:** For those allocated to CRT treatment , the mean number of days in hospital over six months was 25.6 and for those allocated to standard care it was 43.1 days (p =0.01).  **Satisfaction:** Satisfaction interviews with 97 of the participants (response rate = 39%) after 8 weeks found that satisfaction was significantly higher in the CRT cohort than in the control group (26.6 out of 32 for the CRT group, v 22.1 for the control group; p =0.003) |
| Keown (2007) [45] | The CRT is based on the model described by Hoult (1986). It runs alongside an Assertive Outreach Service' | Acute inpatient service + non-acute community mental health teams | **Service use:** Mean weekly admission rates fell from 24.5 (SD 4.0) in 2000 to 14.1 (SD 3.7) in 2004 (ANOVA, f=10.1, d.f.=4, p<0.0005). Occupied bed days did not reduce significantly. They only fell by 22% because length of stay increased. The median steadily increased from 15.5 days in 2000 to 25 days in 2004 (Kruskal-Wallis test, x2=14.6, p=0.0005).  **Other outcomes:** The number of suicides remained constant |
| Kolbjornsrud (2009) [46] | Psychiatric outpatient wards receiving crisis team assistance from clinical staff who assist in emergency situations by providing advice by telephone; providing assessments and follow-ups; and assisting with hospital admissions | Municipalities receiving support from an adult acute psychiatric outpatient unit, but with no crisis team | **Service use**: Yearly admission rate for admissions to the psychiatric inpatient department were reduced by 25% with a CRT, compared to a 13% reduction for the catchment area without a CRT |
| Piggott (1993) [47] | Clients are seen within one hour of referral. On-going at-home and office-based behavioural health services are offered to teach patient and family how to manage and solve problems with living at home. There is a same day psychopharmalogical appointment with a psychiatrist | Acute inpatient services: no details provided about community services | **Service use:** The CRT averted hospitalisation for 151 of 187 clients (80.7%) referred during the study period. 100% in the comparison service were hospitalised.  149 (45.9%) non-CRT patients were readmitted compared with only 22 (11.8%) of CRT patients. The average length of stay for CRT patients who were hospitalised was 7.48 days versus 11.97 days for non-CRT patients. There is no analysis of the data to inform us if these findings are significant or not |
| Tyrer (2010) [13] | The members of this CRT were trained by a long established service in Birmingham and followed the "standard model" of this service |  | **Service Use:** There were no significant findings. In both the CRT and the control there was a non-significant reduction in admissions from the first time period to the second (both 7.7%; p=0.98) and a non-significant reduction in bed days (24.4% CRT, 28.5% control; p=0.56).  **Satisfaction:** There were no significant differences between the services in terms of satisfaction.  **Other outcomes:** There were more compulsory admissions in the CRT (up 31% from first time period) compared with an increase of 7% in the control group from the first to the second period. This finding was not significant. Quality of life and social functioning showed no important differences between the services |

**Table DS12: CRT implementation review - CRTs versus other services: Further details of service characteristics**

| **Study Ref.** | **24 hour service (Y/N/?)** | **Gatekeep all admissions**  **(Y/N/?)** | **Staffing levels>14 staff per 150,000**  **(Y/N/?)** | **Medical cover within team**  **(Y/N/?)** | **Multi-disciplinary team***  **(Y/N/?)** | **Duration of care** | **Early discharge service (Y/N/?)** |
| --- | --- | --- | --- | --- | --- | --- | --- |
| Adesanya (2005) [36] | Y | Y | ? | Y | Y | ? | Y |
| Barker (2011) [12] | Y | Y | N | Y | Y | Mean = 3wks | Y |
| Bechdolf (2009) [37] | Y | Y | N | Y | Y | Mean = 25 days | ? |
| Dean (1993) [38] | Y | Y | ? | Y | ? | ? | ? |
| Dibben (2008) [39] | N | Y | ? | N | Y | Mean = 10.52 days (SD 14.91). | N |
| Forbes (2010) [40] | N | N | Y | Y | Y | Discharged once crisis resolved. | Y |
| Guo (2001) [41] | ? | Y | ? | Y | N | ? | ? |
| Hugo (2002) [42] | N | N | N | Y | Y | ? | ? |
| Jethwa (2007) [43] | Y | Y | N | Y | Y | ? | ? |
| Johnson (2005) [8] | Y | Y | ? | Y | Y | Discharged once crisis resolved. | Y |
| Johnson (2005b) [9] | Y | Y | ? | Y | Y | Discharged once crisis resolved. | Y |
| Johnson (2008) [44] | ? | ? | Y | ? | ? | ? | ? |
| Keown (2007) [45] | Y | Y | ? | ? | ? | ? | ? |
| Kolbjornsrud (2009) [46] | N | Y | Y | Y | N | 2 weeks | N |
| Piggott (1993) [47] | Y | Y | N | Y | Y | 1-2 weeks home treatment. | Y |
| Tyrer (2010) [13] | Y | Y | ? | ? | ? | ? | ? |

**Table DS13: CRT implementation review - Stakeholders’ views on CRTs: themes and recommendations from included studies**

SU=service users; C=carers, MHS=mental health staff, GP=General Practitioner)

| **Area of CRT** | **Theme** | **Description** | **Study reference numbers** | **Stakeholder groups**  **(SU, C, MHS, GP)** |
| --- | --- | --- | --- | --- |
| Continuity within CRT | Continuity of care | CRTs should limit the number of staff working with a service user | [16,17,49,50,51, 55,61,63,65,66] | SU, C, MHS |
|  |  | CRTs should ensure good record keeping and information-sharing between staff | [16,17,49,50,53, 58,61,63] | SU, C, MHS |
|  |  | CRTs should provide information to service users about the CRT (e.g. staff names, contacts) | [17] | SU, C, MHS |
|  | Therapeutic approach | Ensure consistency of therapeutic approach and techniques used by psychiatrists within CRT | [4,17,55] | SU, C, MHS |
|  | Discharge | Clear definition of early discharge and sufficient transition time | [16,49,50,51,55, 61,63] | SU, MHS |
|  | Training | Sufficient formal training for CRT staff | [53,68] | SU, MHS |
|  |  | Sharing experience and information within CRT team | [53,68] | SU, MHS |
|  | Guidelines | No target orientation, but instead qualitative measures of success | [61] | MHS |
|  |  | Constant feedback between research process and practice | [68] | MHS |
| Continuity between services | Integration | Communication and integration with other local mental health services | [4,16,17,49,52,54, 55,58,61,62,63,64,66,68] | SU, C, MHS, GP |
|  |  | Regular meetings between CRT and referrers | [59] | GP |
|  |  | Clear, direct channels of service | [59] | GP |
|  | Eligibility criteria | Clear eligibility assessment rules (less restrictive) | [17,54,55,58,61,62,63,65] | SU, C, MHS |
|  |  | CRTs should not work with service users with emotionally unstable personality disorder, who are very paranoid or acutely manic | [67] | MHS |
|  |  | CRTs should provide a clear definition of what constitutes a crisis for referrers | [67] | MHS |
|  |  | CRTs should not work with SU’s with severe psychosis | [59] | GP |
|  |  | If SU not eligible for CRT, CRT should signpost/refer to appropriate service | [59] | GP |
|  | Gatekeeping | CRTs should have open possibility of hospitalisation to SU, if he/she prefers that and/or it is necessary | [4, 17, 63] | SU, C, MHS |
|  |  | CRTs should gatekeep all admissions to hospital | [4,61,63] | MHS |
|  |  | CRTs/local service systems should have clear guidelines on gatekeeping | [4,63,68] | SU, MHS |
|  | Referrals | CRTs should facilitate appropriate, quick and swift referrals from other services (e.g. GP) | [4,17,49,53,59,61,63] | SU, C, MHS, GP |
|  |  | CRTs should make re-referrals to CRT easy for known clients | [49] | SU |
|  |  | CRTs should supply referrers (e.g. GPs) with information on referred SU’s promptly and regularly (especially after initial assessment) | [59] | GP |
|  | Recruitment | Not taking highly qualified staff from hospitals to CRTs | [55,61,63] | MHS |
|  | Publicity | Inform other services and the public about CRTs | [49,61,63,68] | SU, MHS |
|  | Long-term vs. Short-term | Clear bridge required between short-term, medium-term and long-term services by improving connections between them | [4,17,49,53,59,61,63,68] | SU, C, MHS, GP |
| Team organisation | Staffing | Clear roles within CRT teams and flexible but well-organised shift allocation | [4,53,55,61,62,62,67,68] | SU, MHS |
|  |  | CRT teams need adequate staffing to meet demands (especially out-of hours) | [4,17,53,55,61,63,64,67,68] | SU, C, MHS |
|  |  | CRTs should promote team work and good communication among staff | [4,16,50,61,68] | SU, C, MHS |
|  |  | Communication within all layers of CRT; introduction of CRT representatives committee | [66,68] | SU |
|  |  | Good skill mix in CRT team | [67] | MHS |
|  |  | More use of “homemakers” (support staff) in CRT teams (not just doctors and nurses) | [68] | SU |
|  |  | CRTs should coordinate out of hours crisis response, e.g. With dedicated manager for out-of-hours emergency crisis work | [4,67] | MHS |
|  |  | CRTs should develop clear acute care pathways and protocols, e.g. through the introduction of “pathways development worker” | [4] | MHS |
|  |  | CRTs should include consultant psychiatrists | [4] | MHS |
|  | Other resources | More funding | [4,68] | SU, MHS |
|  | Decision-making | Transparency on executive decisions | [4,55,64,68] | SU, MHS |
|  | Team working Strategies | Regular and frequent meetings of CRT | [16] | SU |
|  |  | “Traffic light system” – cater urgent crises first, medium ones next and less urgent ones last | [16,67] | SU, MHS |
|  |  | Adapt to area (e.g. for rural area: implement mobile and main base site; more staff working fewer hours in larger area; working weekends and flexibly) | [17,4] | SU, C, MHS |
|  | Opening hours | 24/7 telephone service | [16,49,51,55,65] | SU, C, MHS |
|  |  | 24/7 visit and treatment service | [4,16,17,49,61,63,65] | SU, C, MHS |
| Interventions provided | Practical help | CRTs should provide help with everyday tasks (e.g. transport, shopping, hygiene, etc.) | [49,52,55,61,62,63] | SU, MHS |
|  |  | CRTs should support service users with non-treatment-related beneficial activities | [49] | SU |
|  | Quality of service | CRTs should develop and monitor key competencies for staff | [50] | SU, C |
|  | Assessment | CRTs should assess referred SU’s promptly | [54] | C |
|  |  | CRTs should carefully assess single parents with regard to children’s wellbeing and safety | [57] | SU |
|  |  | CRTs should assess carers’ expectations and feeling of ability to cope at general assessment | [54] | C |
|  | Talking support | CRTs should provide staff time for service users to have someone ‘just listen’ | [16,17,49,50,51,5255,62] | SU, C, MHS |
|  |  | CRT staff should provide unrestricted time to talk at start of contact between SU and CRT | [16,17,49,56,68] | SU, C, MHS |
|  |  | CRTs should promote building strong bond with SU’s | [16,56,60,68] | SU |
|  | On-going support | CRTs should offer or arrange follow-up for service users post-CRT discharge | [16,50, 59,65] | SU, C, GP |
|  |  | CRTs should identify a support person post CRT discharge to help prevent relapse | [58] | SU, C, MHS |
|  | Medication | CRT staff should monitor and provide reminders to service users re taking medication | [58] | SU, C, MHS |
|  |  | CRTs should ensure the provision of correct and appropriate medication | [50, 63] | C, MHS |
|  |  | CRTs should not have an exclusive/primary emphasis on medical help | [17] | SU, C, MHS |
|  |  | CRTs should provide very substantial medical input | [4] | MHS |
|  | Information | CRTs should provide appropriate information about illness, crisis, care options, etc. to carers | [16,17,49,50,56,68] | SU, C, MHS |
|  |  | CRTs should provide appropriate information about illness, crisis, care options, etc. to SU’s | [16,17,51,53,56,66,68] | SU, C, MHS |
|  |  | No or little use of medical jargon, explain condition in several angles | [16,56] | SU |
|  |  | CRTs should provide advice over the phone to service users and carers | [17,49,50] | SU, C, MHS |
|  | Involvement | Involvement of carers | [50,68] | SU, C, |
|  |  | Help lower burden for carers | [54,61,63,68] | SU, C, MHS |
|  |  | Give SU’s choice of level of involvement from CRT | [16,17,53,56, 60,68] | SU, C, MHS |
|  |  | Sufficient warning from CRT before changes to treatment and support , explanation of changes and details | [16] | SU |
|  |  | Jointly negotiated crisis plan (& for future crises) | [16,17,68] | SU, C, MHS |
|  | Other | Directing SU’s to other help sources (e.g. GP, support groups etc.) | [49] | SU |
|  |  | CRTs should provide or help service users’ access peer support | [17] | SU, C, MHS |
|  |  | Having host families take on patients for treatment time as alternative or complementation to CRT | [17] | SU, C, MHS |
| Staff Qualities | Attitude | Friendliness, good interpersonal skills | [16,17,49,50,60, 65,66] | SU, C, MHS |
|  |  | No judgement | [16, 17, 49,57, 60,61, 63] | SU, C, MHS |
|  |  | Respectful treatment | [16,17, 57, 66,68] | SU, C, MHS |
|  |  | Ability to remain calm, unstressed and not being phased by SU’s behaviour | [16,56] | SU |
|  |  | Work successfully with service users from different ethnic backgrounds to their own | [69] | SU |
|  | Sensitivity | CRT staff should not focus exclusively on past negative experiences; focus on positive elements of recovery | [49, 61] | SU, MHS |
|  | Mental Health experience | CRTs should recruit staff with personal experience of mental ill health | [17] | SU, C, MHS |
| Treatment at home | Benefits | CRTs should provide home treatment where possible: Familiar surroundings comforting; No other mental health patients around to compare oneself with; avoid unnecessary hospitalisation; safe environment | [17,50, 51,55, 57,59,60,61, 63, 65,69] | SU, C, MHS, GP |
|  | Availability | CRTs should provide immediate treatment during crisis, promptness of response | [16,17,49,50,56, 58 ,59,67] | SU, C, MHS, GP |
|  | Number of visits | CRTs should visit service users frequently | [49,61,63,65] | SU, C, MHS |
|  | Length of intervention | CRTs should remain involved as long as required to ensure recovery from a crisis | [16,49,50,59,66] | SU, C, MHS, GP |
|  | Punctuality | Make an effort to be on time at SU appointments and/or give sufficient warning | [16,49, 50, 63,66] | SU, C, MHS |
|  | Regularity | Ensure regular visits to SU’s | [49,50,63] | SU, C, MHS |
|  | Flexibility | CRT staff and teams must adopt flexible approaches to crises –i.e. be prepared to change plans several times a day and being equipped for that | [49,50,67,68] | SU, C, MHS |
|  |  | Service tailored to SU (pace and situation) | [17,49,67,69] | SU, C, MHS |
|  | Mobility | CRTs should work with a “mobile work base” (i.e. be prepared to work out of the office regularly) | [50] | SU, C |

**Table DS14: CRT implementation review - recommendations from included government and expert guidelines**

| **Area** | **Guidance/Recommendations** | **Source** |
| --- | --- | --- |
| **Team organisation** | | |
| Size | CRT team size approx. 14 with a caseload of 20-30 service users and catchment of 150,000. Areas with higher need will require more staffing. Team should not be smaller than 10 or 11 staff members, with capacity to visit service users at least 2x per day | [2,6,75,76,80] |
| Multidisciplinary/Skills | CRT team should be multi-disciplinary; workers should have a range of skills. There should be 1 full-time administrative assistant  Psychiatrists should help ensure team encompasses full range of skills  All staff should meet essential knowledge and skills laid out In Capable Practitioner Document | [2,6,72,73,80,81] |
| Medication Management | CRTs should appoint staff member to manage medication | [71] |
| **Team roles** | | |
| 24 hour service | CRTs should be available 24/7. There should be a consultant psychiatrist, medical advice, telephone triage and medical prescriber available 24/7 | [6,2,71,73,75,76,78,80,83,84] |
| Gatekeeping | CRTs should act as a gatekeeper | [2, 75, 76, 78, 80, 84] |
| Age | CRTs should serve adults 16-65 and older adults | [2, 70, 78] |
| Service user presentation | CRTs should serve people with primary diagnosis of severe mental illness in an acute episode or crisis (including personality disorder). Can work with people with no previous contact with psychiatric services  CRT services not appropriate for mild anxiety disorders, primary diagnosis of alcohol or other substance misuse, brain damage/dementia, learning disabilities, recent history of self harm but no psychosis, crisis related solely to relationship issues. CRT should be offered as first-line service to support people with psychosis or schizophrenia during an acute episode in the community if severity exceeds the capacity for early intervention services or community teams to manage it | [2, 5, 75, 76, 80] |
| Service Centre | The CRT should have a premises centrally located in the catchment area | [80] |
| Referrals | Referrals to CRTs can be accepted from Accident & Emergency, General Practitioners, and self-referral  CRT to follow clear referral procedure and to feed back to referrer within 24hrs of acceptance of referral  CRT should be the single point of entry to all other acute services | [5, 6, 80, 83] |
| Time | Rapid assessment within 1 hour of referral. Crisis should be responded to within 1 hour; people in crisis should be seen within 4 hours | [2, 76, 78, 80, 83] |
| **Content and Processes of Care** | | |
| Screening/Pre-Assessment | CRTs should conduct an initial screening to determine if the referral is appropriate and urgency of response required. There should be a Pre-Assessment to collect information. There should be clear admission and discharge criteria | [2, 6, 78, 80, 82] |
| Assessment | CRT assessment should include physical health assessment where needed and MDT assessment of service users' level of risk. The initial assessment should be face to face. Focused care plan should be produced involving service user and carer. Assessment should be undertaken by experienced professional competent in crisis working. CRT should work to a protocol for holistic assessment | [2, 6, 83] |
| Medication | Immediate access to supportive medicines is vital. The full resources of a pharmacy able to meet needs of CRT should be available  Medicine charts should be used  Where appropriate non-medical prescriber and patient group directives should be utilised  Administration of medicines should be undertaken by qualified nursing staff only. Where non-qualified nursing staff assist service users to self-administer, this should be supported by local protocols and training  Service users should be given the opportunity to discuss medication options and potential benefits and side effects. There should be monitoring of high doses and metabolic effects of antipsychotic medication | [6, 70, 71] |
| Staff | Mental health teams should "front-load" the patient pathway so that the expertise of the most highly skilled staff is used early on | [70] |
| Team Approach | CRTs should use team approach not key worker roles  Team should adopt a culture of key values  Team workers should work closely and interdependently  Leadership should be based on expertise/competence in specific areas | [74, 76, 77, 80] |
| Intervention | CRT intervention should be intensive, supportive, and encourage active involvement of carers. Problem solving approach, service user strengths and social systems model used  A range of recovery focussed, psychological and evidence based interventions, advocacy, and peer support should be offered  CRT should provide designated named worker who sees the service user at least 2x per week  CRT should be sufficiently resourced to provide continuity of care and effective interventions alongside advice and support | [2, 6, 73, 76, 78, 80], |
| Location | Treatment should be provided in the least restrictive environment, at home where possible. Telephone and face-to-face counselling should be offered | [2,80] |
| Time | CRT should provide intensive contact over short time (usually 2-3 weeks) Patients often seen once per shift. Home treatment visits should be frequent (up to 3 times per day). Should be extended time available for assessment and intervention | [2, 73, 76, 80] |
| Discharge/Relapse Prevention | CRTs should stay involved until the crisis is resolved and service user linked to on-going care. Discharge planning should begin from first contact. Emphasis placed on relapse prevention strategies | [2, 5, 73, 76, 78, 80] |
| Referral to other services | CRT visits need to be down to 1-2 per week before it is reasonable to transfer to a CMHT. If hospital treatment required, CRT should actively plan discharge. CRT should have an awareness of physical health needs that could be better treated in hospital | [6, 78] |
| Risk | | |
| Risk policy | CRTs have risk assessment and management plan in place that that supports positive risk taking  CRTs have written risk policy  CRTs have personal safety protocols for staff | [2, 75, 76] |
| Caseload | CRTs should have a shared caseload. Decisions regarding risk should be made as a team when possible | [76] |
| Risk information | CRT should work with acute care units and recovery houses to share information on risks and needs | [5] |
| **Training & Supervision** | | |
| Supervision | All staff should have quality clinical supervision and daily meetings | [6, 76, 80] |
| Training | All staff should receive on-going training. This should include training in risk management, social systems approach, health belief models, MDT, and a non-medical prescriber course  All staff should be trained to the NMC standards in the clinical, practical, and legal use of medicines  CRT and acute inpatient service staff training should be integrated  CRT Psychiatrists should be involved in training other staff  Access to Patient Group Directives should be increased through more training to develop nurses | [71, 73, 75, 76, 78, 80] |
| **Evaluations and Monitoring** | | |
| Content and process of monitoring | CRT audits should include number of referrals, assessments, home treatment vs. hospitalisation, source of referral, problem/diagnosis of individual, service user and carer experience data and satisfaction, response times, medication, data quality on ethnic group, data completeness of the mental health minimum data set and influences of the rest of the local service system  CRT’s recovery focus should be demonstrated by outcome measures  CRTs should have access to pharmacy and medical colleagues who should oversee audits | [2, 6, 69, 70, 75, 85] |
| **Service User and Carer Involvement** | | |
| Carer’s role | Carer and role they play identified at first contact, or as soon as possible thereafter | [82] |
| Care-giving choice | CRT staff should give carers a choice in whether they continue to provide support to the service user receiving CRT. If carer can no longer provide care, staff should offer realistic alternatives | [2,79] |
| Carer’s assessment | CRTs should provide a carer's assessment where needed, but only if the information is used to determine what support is needed and action is taken  CRTs should clarify carer’s view of the crisis, the implications for them and how they might be involved in resolving it | [6, 76, 79] |
| Respect and support for carer | CRT staff should listen to and value the carer, be available to talk to him/her, treat him/her with courtesy and respect. Staff are ‘care aware’ and trained in carer engagement strategies, and consider and meet the support needs of the carer | [79,82, 83] |
| Social network | CRT should spend time talking to service users and their social networks | [76,81, 83] |
| SU views respected | Service users’ personal views/preference should be considered when making decisions regarding admission to Acute Services vs. CRT | [78] |
| Information | Service users and carers should be given clear information on CRT services, respite resources, who keyworker and GP are, their rights under the Mental Health Act and Mental Capacity Act, and educated about when to call for help | [2, 6, 78, 83] |
| Carer’s training | Leadership from CRT is required to develop training packages for carers who are involved in managing medicines for people in crisis | [2,71] |
| Confidentiality | Policy and practice protocols re confidentiality and sharing information are in place | [82] |
| Responsibility for carers | Defined post(s) responsible for carers are in place | [82] |
| Information for carers | A carer introduction to the service and staff is available, with a relevant range of information across the acute care pathway | [82] |
| Carer support services | A range of carer support services is available | [82] |
| **Working with other services** | | |
| Care pathways | Acute care pathways should be developed and agreed locally | [6, 75] |
| GP referral screening | CRTs may need a screening process for GP referrals to prevent inappropriate referrals | [75] |
| Early discharge | The CRT should communicate effectively with inpatient services to assess service users for early discharge | [75,76, 84] |
| A&E | Accident and Emergency should have their own psychiatric liaison, including out of hours service. A CRT must avoid excessive referrals from A&E. CRTs should not default out of hours work to A&E | [75, 76, 78] |
| Respect for referrers | In order to support effective working with other services, CRT staff should be polite, helpful, and responsive to referrers | [76] |
| Work with care coordinators | CRT staff should secure the involvement of care-coordinators from the other teams such as the CMHT during the time that they are working with the service user | [6, 76] |
| Inter-team meetings | Frequent meetings are needed between the CRT and other mental health teams for case discussions, to build effective working relationships and ensure a dynamic system | [76, 78] |
| CMHT working agreement | An agreement should be reached with the CMHT that priority will be given to service users referred from the Acute Inpatient Unit and CRT | [76] |
| A&E working agreement | Liaison agreements with A&E departments are crucial | [78] |
| Shared work | Shared managerial and administrative functions across community teams reduces inefficiencies created by multiplicity of community teams | [70] |
| CRT and inpatient co-location | There should be regular dialogue between CRT and inpatient teams regarding referrals, admissions, and discharges. This may occur by co-locating CRT and inpatient teams on the same site | [73] |
| Joint roles | Service managers should consider the use of staff rotation and joint roles for acute care and CRT staff and managers between teams | [73] |
